# “Midwives do not appreciate pregnant women who come to the maternity with torn and dirty clothing”: institutional delivery and postnatal care in Torit County, South Sudan: a mixed method study

**DOI:** 10.1186/s12884-020-02910-2

**Published:** 2020-04-28

**Authors:** Pontius Bayo, Loubna Belaid, Elijo Omoro Tahir, Emmanuel Ochola, Alexander Dimiti, Donato Greco, Christina Zarowsky

**Affiliations:** 1 Department of Obstetrics and Gynecology, Torit State Hospital, Torit, South Sudan; 2 Department of family medicine, McGill University, Montreal, Canada; 3 State ministry of Health, Imotong state, Torit, South Sudan; 4 Department of public health, St, Mary’s Hospital Lacor, Gulu, Uganda; 5 Ministry of health, Juba, South Sudan; 6 School of public health, University of Rome, Rome, Italy; 7 School of Public Health, Montreal University, Montreal, Canada

**Keywords:** Maternal health, Institutional delivery, Postnatal care, Determinants of access to health care services, Quality of care, Respectful care, In- kind payments, Social stigma, South Sudan, Mixed methods

## Abstract

**Background:**

South Sudan has one of the highest maternal mortality ratios in the world, at 789 deaths per 100,000 live births. The majority of these deaths are due to complications during labor and delivery. Institutional delivery under the care of skilled attendants is a proven, effective intervention to avert some deaths. The aim was to determine the prevalence and explore the factors that affect utilization of health facilities for routine delivery and postnatal care in Torit County, South Sudan.

**Methods:**

A convergent parallel mixed method design combined a community survey among women who had delivered in the previous 12 months selected through a multistage sampling technique (*n* = 418) with an exploratory descriptive qualitative study. Interviews (*n* = 19) were conducted with policymakers, staff from non-governmental organizations and health workers. Focus group discussions (*n* = 12) were conducted among men and women within the communities. Bivariate and multivariate logistic regression were conducted to determine independent factors associated with institutional delivery. Thematic analysis was undertaken for the qualitative data.

**Results:**

Of 418 participants who had delivered in the previous 12 months, 27.7% had institutional deliveries and 22.5% attended postnatal care at least once within 42 days following delivery. Four or more antenatal care visits increased institutional delivery 5 times (*p* < 0.001). The participants who had an institutional delivery were younger (mean age 23.3 years old) than those who had home deliveries (mean age 25.6 years). Any previous payments made for delivery in the health facility doubled the risk of home delivery (*p* = 0.021). Women were more likely to plan and prepare for home delivery than for institutional delivery and sought institutional delivery when complications arose. Perceived poor quality of care due to absence of health staff and lack of supplies was reported as a major barrier to institutional delivery. Women emphasized fear of discrimination based on social and economic status. Unofficial payments such as soap and sweets were reported as routine expectations and another major barrier to institutional delivery.

**Conclusion:**

Interventions to stop unofficial payments and discrimination based on socio-economic status and to increase access to ANC, delivery services and PNC are needed.

## Background

Globally, maternal and neonatal health have improved significantly with the average Maternal Mortality Ratio (MMR) falling from 385 deaths per 100,000 live births in 1990 to 216 in 2015, corresponding to a decline of about 44% in numbers of reported deaths [1]. However, the MMR remains much higher in Sub-Saharan Africa at 546 deaths per 100,000 live births. This represents 66.3% of global deaths [1]. South Sudan contributed disproportionately to these deaths with an unacceptably high MMR of 789 per 100,000 live births as the country entered the era of Sustainable Development Goals (SDGs) [1].

The majority of these deaths result from obstetric complications that occur during labor and delivery or during the immediate postnatal period [2, 3]. Skilled care during labor and delivery and in the immediate postnatal period has been recommended as a key intervention for preventing these deaths [4]. While “skilled care during labor and delivery” is not synonymous with “facility delivery”, major efforts at improving maternal, newborn and child health over recent years have emphasized a dual approach of improving the technical and human quality of care in health facilities and actively encouraging women to deliver in health facilities rather than at home and attended to by Traditional Birth Attendants (TBAs). However, access to skilled care during labor and delivery remains low in many sub-Saharan countries. The prevalence of health facility deliveries across sub-Saharan Africa increased from 44% in 1990 to only 57% in 2014 [5]. In South Sudan, only 11% of deliveries are known to occur in health institutions according to the 2010 South Sudan household survey data and of these, only 19.4% are attended to by skilled personnel [6].

The way women and their families perceive the benefit and/or the need for an institutional delivery with skilled attendance influences their decision- making process to seek skilled care for either a routine delivery or a complication during delivery. This perception is in turn influenced by their general awareness of the risks of labor and delivery and their capabilities, in terms of material resources, to access health care [7]. The strong correlation between health institution delivery and mother’s education and wealth quintile has also been well documented in the literature on Sub-Saharan Africa [8]. However, institutional deliveries need to be planned for well in advance. This “birth preparedness” includes saving money, identification of a health facility and a skilled care provider, identifying transport and making other preparations to reduce delays in reaching care [9]. Such arrangements also facilitate emergency care should complications occur [10].

South Sudan has been through protracted civil wars for over two decades. These have disrupted social and community structures and have seriously damaged the health system [11]. The conflict is said to have disproportionately affected the health of women and children [12]. Health data crucial for determining the changing public health needs and priorities of women and their children are limited. There is in particular a paucity of evidence on the utilization of health institutions for deliveries and postnatal care. Three recent publications report low rates of deliveries by skilled birth attendants as well as a range of reasons for high rates of home deliveries: a cultural preference for home deliveries and lack of confidence in health facility capacities attributed to lack of skilled staff [13]; socio-economic barriers to institutional delivery including demands for payments by health facilities, long distances and insecurity along the road [14]; and men, who were the main decision makers regarding the place of delivery, preferring that their partners deliver at home [15]. As part of a larger intervention and implementation science project to improve maternal and neonatal health in Torit County, South Sudan and Gulu District, Uganda, the study reported here focused on understanding the dynamics of health institution utilization for deliveries and postnatal care in a conflict affected setting.

According to one recent analysis, 50% of the population in Torit County is within five kilometers or one hour’s walk of a public health facility [16]. However, the current patterns and variation of utilization of these facilities for routine delivery care and the factors that influence their use for deliveries are not well documented. We sought to narrow this evidence gap to inform an intervention to improve maternal and neonatal health in Torit County in South Sudan. The aim was to determine the prevalence and explore the factors that affect utilization of health facilities for routine delivery and postnatal care.

## Methods

### Study design

A convergent parallel mixed method design [17] comprising a community based- cross sectional survey and an exploratory qualitative study was used. Equal importance was given to qualitative and quantitative data which were combined in the interpretation phase. The study was conducted between March and December, 2016.

### Conceptual framework for the qualitative study

To explore the barriers for accessing maternal health services, the Gabrysch and Campbell framework [10] rooted in the three delays model [18] was used. The framework addresses four dimensions: *socio cultural factors* (traditional beliefs, norms, gender dynamics), *perceived needs and benefits* (knowledge, information, perceived quality of care), *economic factors* (ability to pay) and *physical accessibility* (distance, transport, roads).

### Study setting

The study reported here is part of a larger research project supporting participatory interventions with women and communities on the one hand and health workers and facilities on the other in two neighboring conflict-affected settings: Torit, South Sudan and Gulu, Uganda. The project was initiated by the staff of two hospitals with some existing links; St Mary’s Lacor Hospital in Gulu and Torit State Hospital in South Sudan. Both hospitals had played central roles in maintaining some health care over decades of war, and both sought to engage more effectively and responsively with community priorities and to learn from and with the other setting’s experiences.

The study was conducted in two payams of Torit County in Imotong state, Republic of South Sudan: Nyong and Himodonge. Payams, in South Sudan, are administrative areas of at least 25,000 residents. Several payams constitute a county which in turn constitute a state. Payams are themselves composed of smaller administrative units called bomas. Each boma includes several small villages or hamlets.

Nyong and Himodonge are among the eight payams of Torit County. Each has five bomas. Their total projected population was 61,297 in 2016 with 2212 pregnancies expected annually [19]. Nyong Payam in which the state capital, Torit, is located was the most populated with 49,419 inhabitants while Himodonge Payam had 11,878 residents. Torit town is located in south eastern South Sudan, about 150 km from Juba, the capital city of South Sudan. While Nyong Payam is considered urban, there are distinct neighborhoods and villages within the Payam.

There are five public health care levels in South Sudan: Primary Health Care Units (PHCUs), Primary Health Care Centers (PHCCs), County Hospitals, State Hospitals and Teaching Hospitals. PHCUs are the lowest level facilities and provide preventive, promotional and curative services but not delivery services which are offered at all the other levels [16]. At the time of the study there were only three public health facilities above the PHCU level and therefore able to conduct deliveries in the study area.

### Study population and sampling

A sample size of 383 was calculated for a 95% confidence level, using OpenEpi version 3 for sample size calculation for the proportion of women who deliver in the facilities assuming a hypothesized facility delivery rate for South Sudan of 21% [7] and design effect (for cluster surveys) of 1.5, and allowing 10% for missing data.

The survey participants were selected among the women who had given birth in the previous 12 months preceding the survey. A four-stage stratified sampling technique was used to select the participants. It was designed to capture urban and rural populations and people living at varying distances from a health facility staffed by skilled birth attendants. First, three out of the eight payams of Torit County (Kudo, Nyong and Himodonge) were purposively selected for the study. Nyong is an urban payam, Himodonge is a rural payam, and Kudo is a remote payam, about 2 h outside of Torit town during the dry season and often inaccessible during heavy rains. Kudo was excluded at the time of the survey because of insecurity resulting from renewed open conflict in the area in July 2016. In the second stage, three bomas out of the five in each payam were selected: the one closest, at middle distance and farthest from the facility offering skilled birth attendance. These distances were about 0.5, 2 and 6 km respectively for the bomas in Nyong Payam and 0.5, 6 and 26 Km for those in Himodonge Payam. In the third stage, three villages or hamlets were selected by simple random sampling from a list of the villages in each selected boma. On the interview day, vehicles dropped interviewers at an estimated geographical central location of the village, from which interviewers used chain or sequential referral technique [20] and home health promoters to identify the next household that qualified for the interview and seeking to capture all or most women normally residing in that village or hamlet who had delivered in the previous 12 months. Home health promoters in South Sudan are community members identified by the communities and supported by the government to promote community health and facilitate linkage to the health facilities.

### Participant selection for the qualitative data

For the qualitative study, five categories of participants bringing diverse perspectives and experiences to the study topic were identified for purposive sampling [21]: (1) policy-makers from State Ministry of Health (SMoH) and the national MOH, (2) staff of NGOs and humanitarian partner organizations, (3) health managers (county health department officials and director of hospital), (4) health workers (physicians, midwives, nurses, clinical officers), and (5) community members including women, men and community leaders.

In consultation with the local research team, a list of participants for IDIs was prepared including policymakers, health managers and NGO staff; and interviews were scheduled. FGDs were also planned at the community level in consultation with the village chiefs and HHPs who helped to mobilise the communities. FGDs with the healthcare providers were planned in consultation with the facility in-charges.

The sample size was not predetermined but rather sought to achieve data saturation. In total, 19 in depth interviews and 12 Focus Group Discussions (FGDs) were conducted (see Table 1).

### Data collection methods

A pretested, standardized adapted survey tool for maternal and child health (MCH) care (from University College London (UCL)) (Additional file 1) was used with permission of UCL and administered by trained data collectors to obtain the quantitative data. Community entry was enhanced by working with home health promoters as guides. Prior to the administration of the questionnaire, informed consent was obtained. Individual interviews were conducted in the home of the respondent in a convenient location offering privacy.

**Table 1:** Number of interviews and FGDs per group of participants

**Data collection Methods**	**In depth-Interviews**	**FGDs**
Communities	0	8 (8–15 participants each)
Health providers (nurses, midwives, clinical officers)	0	3 (8–10 participants each)
Health managers (County Health department, Director Hospital)	4	0
Staff of NGOs and implementing partners	8	0
Policy makers (SMOH, MOH, members of parliament)	7	1
**Total**	19	12

Demographic information was collected on women who had given birth in the previous one year; then questions on socio-cultural, economic and physical barriers that affected the decision making to access care for routine delivery and postnatal care were asked, together with questions on perceptions of benefit or need to seek care.

To gather qualitative data, in- depth interviews and FGDs were conducted to explore the determinants influencing utilization of health services for institutional deliveries and postnatal care. While individual interviews and FGDs with policy makers, health managers, staff of humanitarian partner organizations and health workers were conducted in English, FGDs with the community members were conducted in the local language (Lotuko) by local research assistants familiar with the social and cultural context of the study area. The local research assistants included two women and one male. All of them were trained in basic qualitative research methods by an anthropologist and public health specialist (LB) who has worked with the team and in Torit County since 2015.

The individual interviews were conducted by the research team members who are co-authors of this paper (CZ, EO, LB).

Interview guides for FGD and in- depth interviews were designed according to the research objectives and the conceptual framework. FGDs were recorded and notes were taken systematically during the in -depth interviews as initial attempts to digitally record interviews were resisted by some respondents. The duration of the interviews and FGDs was between 45 min and one hour**.**

### Data analysis

Quantitative data were entered using Epi data software and analysis done using SPSS version 21. Categorical data were summarized into proportions and comparisons made using chi square tests. For continuous data, the mean or median and interquartile ranges were calculated. Bivariate analysis was conducted to assess the association between the place of delivery and the socio-cultural, economic and physical variables as well as perception of need or benefit for institutional delivery. Correlations between different age groups and different payams were assessed using Student’s t test. Significant factors on bivariate analysis were entered into a multivariate logistic regression model to determine the independent variables associated with institutional delivery. The confidence level was set at 95% and all statistical tests were considered significant at a *p*-value ≤0.05.

In depth interviews and FGDs were transcribed and translated from the local language to English by local research assistants. Both were coded with QSR International’s Nvivo software version 11.3.2. The categories and sub categories of themes were organized according to the framework, the research objectives and emergent themes from empirical data. A mixed thematic (inductive/ deductive) approach was used to analyse the data [22]. Data sources (health providers, managers, policy makers, communities) and data collection methods (interviews and FGDs) were triangulated to enhance the internal validity of the study [23, 24].

### Ethical considerations

Ethical approvals for this study were obtained from the ethical committee of the Ministry of Health, Republic of South Sudan and the University of Montreal Hospital Research Centre (CRCHUM, Canada). Informed consent was obtained from all participants. Permission to record the FGDs was sought. Confidentiality and anonymity of the participants were maintained throughout the study process. Data were kept under lock and key only accessed by the research team. We explicitly adopted the guidelines proposed for qualitative research in conflict-affected settings by the ReBUILD Consortium [25]. In particular, these guidelines caution that audio or video-recording interviews and FGDs can sometimes be problematic both scientifically (through limiting the open expression of perspectives) and ethically (through creating a fear of possible reprisals should the recordings be shared) where trust has been eroded in conflict-affected settings.

## Results

Four hundred and eighteen mothers who had delivered in the previous 12 months participated in the survey, 229 from Nyong Payam (representing 59% of the births that would be expected in the 6 selected villages if population were evenly distributed across the 14 villages of the payam) and 189 from Himodonge Payam (140% of expected births if population and births were evenly distributed across the 8 villages). Table 2 shows the socio-demographic and clinical characteristics of the participants. The participants were between 11 and 40 years of age; the mean age was 24.8 years with a standard deviation (SD) of 6.2 years, 35.2% of the participants did not know their age while 34.2% were from 21 to 30 years of age. The mean parity was 3.8 and ranged from 1 to 17. The majority of the participants were of the Lotuko ethnic group (82.3%) and belonged to the Catholic religion (79.4%). In terms of education, 60.5% of respondents had no formal education while 30.1% had completed some primary education and only two (0.5%) participants had reached tertiary level. The majority of the participants (73.2%) had attended ANC at least once, 46.4% of whom attended at least four times. Only 27.7% delivered in the health facility and 1.9% were delivered by caesarean section. Less than a quarter of the respondents (22.5%) reported having made at least one visit for post-natal care within six weeks of delivery and 12.8% of these women made their first visit only after developing a complication.

**Table 2:** Socio-demographic and clinical characteristics of participants who had institutional deliveries compared with those who had non-institutional delivery

**Characteristic**	***N*(%)**
**1. Age (years)**	
≤ 20	84 (20.1)
21–30	143 (34.2)
> 30	44 (10.5)
Don’t know	147 (35.2)
Mean age	24.8 (SD = 6.2)
Range	11–40
**2. Parity**	
1	65 (15.6)
2–5	230 (55.0)
> 5	86 (20.6)
Missing	37 (8.7)
Mean	3.8 (SD = 2.5)
Range	1–17
**3. Payam of residence**	
Nyong	229 (54.8)
Himodonge	189 (45.2)
**6. Level of School attendance**	
None	256 (60.5)
Primary	126 (30.1)
Secondary	28 (6.7)
Tertiary	2 (0.5)
Missing information	9 (2.2)
**7. Antenatal care (ANC) attendance at least once**	
Yes	306 (73.2)
No	100 (23.9)
Missing	12 (2.9)
**8. Place of delivery**	
Home	287 (68.7)
Health facility	116 (27.7)
Missing information	15 (3.6)
**9. The principle person who conducted the delivery**	
Friend/ Relative/ Neighbor	165 (39.5)
TBA	108 (25.8)
Village doctor/ Traditional herbalist	5 (1.2)
Self	9 (2.2)
Doctor	14 (3.3)
Nurse midwife	99 (23.7)
Other	6 (1.4)
Missing information	12 (2.9)
**9. Mode of delivery**	
Normal vaginal delivery	390 (93.3)
Vaginal delivery with vacuum extraction	3 (0.7)
Delivery by caesarean section	8 (1.9)
Missing information	17 (4.1)
**10. Postnatal care (PNC) attendance at least once**	
Yes	94 (22.5)
No	309 (73.9)
Missing information	15 (3.6)
11. Reason for first PNC visit	
For a problem	12 (12.8)
Just for a check	60 (63.8)
Not sure	22 (23.4)

Table 3 below shows a comparison of the characteristics of participants who had institutional delivery with those who had non-institutional (home) delivery. Participants from Nyong Payam were more likely to have delivered in the health facility than those from Himodonge Payam (OR 3.8, 95% CI 2.8–4.8, *P* value < 0.001). Only four mothers (2.1%) out of 189 participants from Himodonge Payam had delivered at the health facility compared to 53.3% for Nyong Payam. The participants who had institutional deliveries were significantly younger (mean age 23.3 years SD 5.3 years) compared to those who had non-institutional deliveries (mean age 25.6 years SD 6.3 years) (*p* = 0.004). However, women who did not know their age tended to have non-institutional deliveries (*p* < 0.001).

**Table 3:** Characteristics of Institutional deliveries compared with non-institutional deliveries

**Characteristic**	**Institutional delivery *n*(%)**	**Non-institutional delivery*n* (%)**	**OR**	**95% CI**	***P*Value**
1. **Payam of residence**					
Nyong	112 (96.6)	117 (38.7)			
Himodonge	4 (3.4)	185 (61.3)	3.8	2.8–4.8	< 0.001
2. **Mother’s age (years)**					
≤ 20	42 (36.2)	42 (13.9)			
21–30	51 (44.0)	92 (30.5)			
≥ 31	13 (11.2)	31 (10.3)			
Unknown	10 (8.6)	137 (45.4)			< 0.001
Mean age	23.3 (SD = 5.3)	25.6 (SD = 6.3)			0.004
3. **Parity**					
1	25 (22.7)	40 (14.8)			
2–5	66 (60.0)	164 (60.5)			
> 5	19 (17.3)	67 (24.7)			0.089
Mean parity	3.2 (SD = 2.2)	3.6 (SD = 5.3)			0.244
4. **Attended ANC at least once**					
Yes	113 (97.4)	193 (66.6)			
No	3 (2.6)	97 (33.4)	2.9	1.8–4.1	< 0.001
Missing information	0	12			
5. **No. of ANC visits for those who attended (*n*** **= 306)**					
1–3 times	32 (19.5)	132 (80.5)			
≥ 4 times	81 (57.0)	61 (43.0)	2.9	2.0–4.1	< 0.001
6. **Education level of mother**					
Did not go to school	31 (26.7)	222 (75.8)			< 0.001
Primary	60 (51.7)	66 (22.5)			< 0.001
Secondary	23 (19.8)	5 (1.7)			< 0.001
Tertiary	2 (1.7)	0 (0)			
Missing information	0	9			
7. **Whether she wanted the pregnancy**					
Yes at the time	39 (34.2)	131 (45.0)			
Yes but later	72 (63.2)	154 (52.9)			
No	3 (2.6)	6 (2.1)			0.136
Missing information	2	11			
8. **Whether husband wanted pregnancy**					
Yes at the time	58 (51.3)	136 (46.9)			
Yes but later	54 (47.8)	151 (52.1)			
No	1 (0.9)	3 (1.0)			0.725
Missing information	3	12			
9. **Suffered complication during pregnancy**					
Yes	33 (28.4)	43 (15.3)			
No	83 (71.6)	238 (84.7)	2.2	1.3–3.7	0.003
Missing information	0	21			
10. **Planned person delivered her**					
Yes	15 (13.2)	101 (36.1)			
No	8 (7.0)	93 (33.2)			
Made no plans	91 (79.8)	86 (30.7)			< 0.001
Missing information	2	22			
11. **Payments made for delivery service**					
Yes, in cash & kind	8 (7.0)	12 (4.3)			
Yes, in kind	70 (61.4)	62 (22.3)			
Yes, in cash	1 (0.9)	36 (12.9)			
No, gave nothing	35 (30.7)	168 (60.4)			< 0.001
Missing information	2	24			
12. **Main decision maker on place of delivery**					
Self	68 (61.3)	105 (37.2)			
Self and Husband	18 (16.2)	113 (40.1)			
Husband alone	13 (11.7)	45 (16.0)			
Mother in-law	5 (4.5)	15 (5.3)			
Father in-law	3 (2.7)	0 (0)			< 0.001
Other	4 (3.6)	4 (1.4)			
Missing information	5	1			
13. **Any complications during labor**					
Yes	18 (16.2)	22 (8.1)			
No	93 (83.8)	230 (91.9)	2.2	1.1–4.3	0.03
Missing	5	30			
14. **Postnatal care attendance at least once within 42 days**					
Yes	47 (40.5)	47 (16.4)			
No	69 (59.5)	240 (83.6)	3.5	2.1–5.6	< 0.001

Attending school to even primary level was significantly associated with institutional delivery compared to not going to school at all (*p* < 0.001). Whether or not the woman or the husband wanted the pregnancy at the time of conception did not affect the place of delivery. Attending skilled ANC at least once was significantly associated with institutional deliveries compared to not attending at all (OR = 2.9, 95% CI = 1.8–4.1, *p* < 0.001). Women who reported having suffered a complication during pregnancy (such as high fever, severe headache, seizures, vaginal bleeding, foul smelling vaginal discharge) were more likely to have an institutional delivery than women who did not (OR = 2.2, 95%CI = 1.3–3.7, *p* = 0.003). Developing a complication during labor (such as high fever, vaginal bleeding, foul smelling vaginal discharge, seizures, retained placenta, prolonged labor, prolapsed umbilical cord) was also significantly associated with institutional delivery (OR = 2.2, 95% CI = 1.1–4.3, *p* = 0.03).

Women who had a non-institutional delivery were more likely to be assisted by a person they had previously planned for compared to women who had an institutional delivery. In fact, women who had institutional deliveries had no plans for who would assist them during delivery (*p* < 0.001). Women were more likely to deliver in the health facility if they independently made the decisions about delivery themselves than if the husband or other relative was involved (*p* < 0.001). Women who had institutional deliveries made more payments (especially unofficial payments) for the service than those who had non-institutional deliveries (*p* < 0.001).

Women who had non-institutional delivery were less likely to attend postnatal care services compared to those who had institutional delivery (OR = 3.5, 95% CI = 2.1–5.6, *P* < 0.001).

On logistic regression analysis, only four factors were independently associated with institutional deliveries: maternal age, four or more ANC visits, payment for deliveries and the payam of residence (Table 4). Mothers who had attended ANC at least four times were five times more likely to have institutional delivery than those who did not attend (95% CI = 0.01–5.13, *p* = 0.007). The younger women also had a 10% higher chance of delivering in the health facilities than their older counterparts (95% CI = 1.02–1.196; *p* = 0.01). Previous payments made for delivery in the health facility increased the risk of home deliveries by about 30% (95% CI = 0.13–0.85; *p* = 0.021) and mothers who resided in Nyong payam had a 4 % greater chance of delivering in the health facility than those residing in Himodonge Payam (95% CI = 0.004–0.374, *p* = 0.005).

**Table 4:** Multivariate logistic regression analysis for independent predictor variables for home deliveries in Torit County

**The predictor variable**	**Adjusted OR**	**95% CI**	***P*value**
Age	1.11	1.02–1.196	0.01
ANC visit at least once	0.01	0.0–7.98	0.168
Four or more ANC visits	4.9	0.01–5.13	0.007
Complication during pregnancy	1.45	0.51–4.121	0.485
Complication during labour	6.33	1.30–30.9	0.023
Planned person for delivery	5.31	1.72–16.32	0.004
Main decision maker on place of delivery	1.2	0.9–1.8	0.150
Paying for deliveries	0.34	0.13–0.85	0.021
Maternal education	1.10	0.00-	0.999
Payam of residence	0.04	0.004–0.374	0.005

**Table 5:** Synthesis of perceptions of barriers for accessing maternal health services

Types of barriers	Empirical data
**Socio cultural factors**	- Policy makers, health providers: Desire to deliver at home (privacy), prefer going to traditional healers, TBA
	- NGO staff: women lack time, too many responsibilities
	- Communities: No social cultural factors were raised
**Perceived needs and benefits**	- Policy makers, NGO staff: lack of health knowledge on the importance of delivering in a health facility
	- Communities: Perceived poor quality of care (poor attitude, discrimination based on social economic status, lack of skilled health care providers, lack of drugs)
**Economic accessibility**	- Communities: Inability to pay for drugs, sweets, soaps and new clothing
**Physical accessibility**	- All categories of participants: distance, insecurity, poor roads, lack of means of transport

In summary, only 27.7% of the mothers in the survey area delivered in the health facility while this figure was only 2.1% for the women in Himodonge Payam compared to 53.3% in Nyong Payam. Only 22.5% of the respondents made at least one visit for post-natal care within six weeks of delivery. Young age, four or more ANC visits and residing in Nyong Payam were the independent factors associated with increased institutional deliveries while any form of payment for delivery services reduced the chance for institutional deliveries.

The results from the qualitative study are presented based on Gabrysch and Campbell’s framework [10] and a synthesis of these perceptions is presented in Table 5 below.

### Perceptions of barriers to maternal health care services utilisation

#### Socio-cultural factors

Policy makers, health workers and NGO staff emphasised cultural norms such as the preference of going to traditional healers until there are serious complications, as well as the privacy of delivering at home, lack of time and high illiteracy as the main barriers to institutional delivery.

*“Women are often not responsive to calls because of other family responsibilities. They never have adequate time. Most of pregnant women prefer the privacy of their homes and want to deliver at home, they fear coming to the health facility” (IDI male, NGO staff).*

*“Communities rely very much on witch doctors but if they failed, then they start running to the hospital and this makes things difficult” (FGD, health workers, Torit state hospital).*

*“Our community attitude is not also good. Some of our people are still going to the witchdoctors, and we need to discourage this. There is also high illiteracy rate contributing to such issues but the educated class are aware of the importance of health. Many women want to deliver at home rather than in the hospital and they come to hospital after a lot of delay at home or if they failed to deliver as expected. There is also lack of health education, people have just come from war, and radio talk shows may help*” (FGD, *policy makers*, *Woman, Torit*).

In contrast, women and men in the communities did not report socio-cultural factors or preferences as barriers to institutional delivery.

### Perception of needs and benefits

#### Lack of knowledge about maternal health and perceived poor quality of care

While policy makers and NGO staff indicated that lack of knowledge regarding maternal health is an obstacle to use of health services, the communities emphasized the poor quality of care received at health facilities as the major obstacle.

*“They lack exposure or awareness concerning antenatal care” (IDI, Male NGO staff, Torit).*

*“Ignorance in the community about the importance of reproductive health is important” (IDI Women, Health manager SMOH, Torit).*

Community members were particularly concerned about the poor attitude of health workers, the lack of skilled health workers and the lack of medicines and supplies in the health facilities. The poor attitude of health workers was reported widely in communities living near the State hospital.

“*The care is poor at the hospital, because we even pay for the stamp*^1^*and payment for examination keeps increasing and sometimes the drugs are not there. Some health workers at the hospital are rough and others are friendly. Those on night duty are always drunk at the hospital” (FGD # 5 Nyong Payam*).

“*The situation is made worse by the hospital charges. In most cases the hospital may not demand money but they indirectly charge in kind. If you deliver in the hospital you have to pay for sweets and soaps which some people cannot afford and so they prefer to deliver from home. If you deliver in the hospital and do not pay for sweets and soaps,*^2^*you will not be discharged; this needs to be stopped” (FGD #4 Nyong Payam)*

*“Sometimes there are drugs and sometimes the drugs are not there in the hospital. The staff on night duty in the hospital do not do their work especially in the admission ward, but the maternity is good. Some of the nurses are harsh with us. Sometimes we receive wrong prescriptions of drugs from staff in the hospital. There is need to recruit well trained and experienced staff at the pharmacy in the hospital. Some staff come for duty while drunk. Sometimes the prescription is done without testing” (FGD #2, Nyong Payam)*

The absence of health workers during night shifts and week-ends was emphasized as an important obstacle to the use of health services by women who live far from health facilities.

*“When pregnant women come to the facility at night, they find no health worker, especially midwives. In case of obstructed labor, if we communicate to Torit hospital there is no immediate response in terms of transport” (FGD #3, Himodonge Payam)*

*“Sometimes, those on duty are not there in the hospital at night and on the weekend. At the maternity ward, midwives asked us to come with soap and sweets and if you failed to bring them after delivery, sometimes they will not discharge you home” (FGD # 1 Nyong Payam)*

Women have also reported social stigma as an important barrier for institutional deliveries:

*“We lack dresses for going to the main hospital in town, so this forces us to deliver at home with the help of our TBA because midwives and doctors at the main hospital do not appreciate pregnant women who come to the maternity with torn and dirty clothing” (FGD #1 Nyong Payam)*

“*Sometimes we do not have good dresses; you cannot go into public with old dirty clothes-----see even the other day a woman’s neighbor had to buy her slippers on the day she was in labor”. “For delivery at home, no one will be there to laugh at you so you can put on your old clothes”.*

*(FGD#4, Nyong Payam)*

#### Physical and economic accessibility

All categories of participants reported insecurity, distance and the lack of transport as important deterrents to access for health care services.

*“Insecurity remains a big challenge and the poor road network is another hindrance” (FGD, health providers, Torit).*

*“We do not have any nearby health facility here at Inna, so we always have to walk a long distance to the hospital. Sometimes we use motorcycles, bicycles or private cars which are very expensive to hire” (FGD #1 Nyong Payam)*

Community members emphasized the lack of ability to pay for care and particularly the unofficial payments that they need to bring to the midwives (soap, sweets). This was identified as the main obstacle for accessing maternal health care services.

*“We cannot afford soap and sweets needed at the maternity of the hospital, and then we prefer to deliver at home” (FGD #2, Nyong Payam).*

*“In the hospital sometimes, they lack drugs and we had to buy the drugs from store in town and they are very expensive. This is a big challenge for us, and also the charges for pregnancy tests are high, it would be better if they can reduce” (FGD #1, communities, Nyong Payam).*

*“We cannot buy bed sheets, soap and good food for ourselves after delivery*” *(FGD#6 Hilieu Himodongue Payam).*

*“It depends but most of them charge four bars of washing soap, four pieces of bathing soap plus 100 SSP (0.5US$)” (FGD #4 communities, Nyong Payam).*

## Discussion

This study has shown that the prevalence of institutional deliveries among the survey participants is 27.7% and that it is only 2.1% for participants from Himodonge and 53.3% for those from Nyong Payam while postnatal care attendance at least once within six weeks after delivery is at 22.5%. This low institutional delivery rate is despite the fact that 73.2% of respondents reported receiving skilled ANC services at least once during the pregnancy. The inability to pay for care, especially unofficial payments, and the perceived poor quality of care were the main barriers to utilizing health facilities for delivery and PNC reported by the women who participated in the study. A recent analysis of 2010 South Sudan Household Survey (SSHS) data found that only 21.1% deliveries occurred in health facilities [26]. Our study shows a slightly higher institutional delivery rate which might be expected as a result of possible improvements in the health system over the years from 2010 following efforts from the government and its development partners. However, the significant disparity between the two payams is of a concern to public health policy. Nyong Payam hosts Torit town, the capital of Imotong state, and has two public health facilities (one of which is a state hospital) able to conduct deliveries compared to Himodonge Payam which is more rural and has only one public health facility, a primary health care centre (PHCC). The population in Nyong Payam may therefore, be more privileged and have easier access to health services than those in Himodonge Payam.

In the qualitative arm of the study, the population from Nyong Payam raised serious issues related to the quality of care offered at the hospital which prevented many of them from using the health services for deliveries and post -natal care, even though the overall rate of facility delivery was higher in Nyong Payam. This finding is consistent with the findings of Gabrysch and Campbell who indicated that some health facilities closer to villages were underutilized because of perceived low quality of care and that the population may prefer traveling longer distance if the health facility has a good reputation [10]. A qualitative study in Zambia and Uganda reported that women would not use health facilities for postnatal care if they had a home delivery due to fear of harassment by healthcare providers [27]. An observational study in other parts of South Sudan showed that healthcare providers offering postnatal care discriminated against the neonates who had been delivered at home by traditional birth attendants compared to those who had institutional deliveries [28]. Perceived poor quality of care has been identified as a major deterrent for accessing health care services in our study, among others [15, 27, 29–33]. This domain was wide-ranging and included specific reference to lack of supplies and drugs, absent health workers, and inebriated health workers, suggesting that indeed “facility delivery” is not yet a good proxy for “skilled birth attendance”. Nevertheless, the responses also reveal that women expect at least adequate technical care from a health facility and that its perceived or experienced absence contributes to choosing a home delivery which also lacks supplies, drugs, and formally trained staff. However, discrimination based on socio-economic status of the women seeking care was particularly emphasized by the participants. This was expressed as the need to buy new clothes so as to appear “socially acceptable”. This finding has been identified in several studies [34–36]. A qualitative study conducted in Southern Tanzania found that if women look “dirty” and poor, they might face humiliation by health providers [36]. This finding was particularly consistent in the context of Tanzania and Ghana [36–39]. Although the literature on the phenomenon of mistreatment and abuse that face women during childbirth in low- income countries is growing rapidly, the literature on effective interventions to address this global health problem is still limited [40, 41].

Regarding the ‘predisposing factors’ (such as maternal age, education level of the mother, parity), ‘enabling factors’ (which included the place of residence) and ‘need factors’ (such as ANC attendance, complications during pregnancy, type of pregnancy- planned or unplanned) our results are consistent with several reviews of literature [8, 10, 31]. Younger age (23 years or less) was significantly associated with institutional deliveries compared with older age. It is possible that young women often carrying their first pregnancies, and perhaps anticipating complications, might prefer to deliver in health institutions. In a mixed methods study in Ethiopia, most women thought it was not necessary to deliver at a health facility after a previous normal delivery [42], yet many of them may be at an increased risk, at times simply because of the high parity as was seen in this sample. Although the younger women tended to report delivering in health institutions in this study, the presence of some very young girls, as young as 11 years, in this sample is of a particular public health concern that needs to be investigated further. It is important to note here that women who did not know their age were significantly more likely to have home deliveries compared to those who knew their age and whether this has a relationship with the education levels of the participants needs further investigation. The education levels among participants in this study were generally low with 90% not having gone beyond the primary level of education; in fact, 60% never went to school at all. Education is known to improve women’s health seeking behavior as it improves their health awareness, economic autonomy and ability to make appropriate health decisions [8, 43, 44]. However, education was not a significant predictor of place of delivery in our study after controlling for confounding factors in multivariate analysis as the sample of participants in this study generally had low education levels. It is worth noting that the protracted civil war has had devastating impacts on the education system and the educational attainment across South Sudan [45, 46].

Attending ANC at least once significantly increased the chances of institutional delivery in our study. This is consistent with findings from other studies and reviews of literature from low income countries [10, 31, 47, 48]. ANC services include health education, counseling, screening for and treatment of complications during pregnancy so as to promote healthy pregnancy experience [49]. ANC visits therefore, link up the families with the health care system and can be a catalyst to increasing the chances of institutional deliveries.

This study also demonstrated that any form of previous payments for delivery services in the health facilities increased the risk of home deliveries. Health services are offered for free in South Sudan in principle but this study demonstrated that the women who had institutional deliveries made unofficial payments and deliveries at home were more likely to be free of charge. The qualitative arm of this study enhances the quantitative findings regarding these unofficial payments as a major deterrent to institutional deliveries. In the context of the study, these unofficial payments are generally sweets and soaps that are expected by health providers from the patients. Culturally, when women deliver at home, the sweets used to be given to the TBA to celebrate the new life that has been given. The soap was also given to TBAs to clean their kits when they were performing the deliveries in villages. These cultural practices have been somehow “transferred” to the health facilities. At the time of the study, health providers working at the hospital were reported to routinely demand such items. Under the care of the TBA, the woman is not in a group of other care seekers and the care she receives is individualized and may not be affected by the type and quantity of sweets or soap given or if she presents nothing at all. In the health facility the situation is different. These unofficial payments are called different names in the literature including “hidden costs” or “informal payments” and are seen as a major obstacle for accessing health care services [31]. Although many countries in Sub-Saharan Africa have removed user fees for different health services especially for Reproductive Maternal Neonatal and Child Health (RMNCH) to meet the goal of Universal Health Coverage (UHC), these informal unofficial payments are still occurring in health systems in many countries [50–54]. The importance of these social and financial barriers is underlined by the finding that women were much more likely to plan and prepare for a home delivery than to prepare and plan for an institutional delivery.

Regarding decision making power on the place of delivery, involving the partners or other relatives was associated with home delivery, though this association was not retained in the regression analysis. This finding is consistent with that in a qualitative study in Kapoeta, South Sudan where men preferred their partners to have home deliveries [15] and suggests the importance of gender and inter- generational dynamics. We are exploring these further in the ongoing research.

In this study, non-institutional delivery translated into non-attendance of PNC services. This finding is similar to findings in Ethiopia where PNC visits were influenced by place of delivery besides skilled care during antenatal period [55]. The interaction with the health care system seems to give the women the incentive to make the postnatal care visits.

The strength of this study lies in its design. The use of a mixed study design has helped to highlight not only the individual socio-demographic factors that influence health institution utilisation for deliveries and postnatal care but also to understand the social and structural factors related the health system. The synergy of the two methods has provided a more comprehensive picture of the determinants influencing the access of health care services. It has highlighted the complexity of the interactions among the different determinants of institutional deliveries.

This study had limitations. First, because the vast majority of deliveries are uncomplicated, the sample size used in this study could not allow for the assessment of benefits of institutional deliveries in terms of clinical maternal and neonatal outcomes. However, a strong inverse relationship between institutional deliveries and maternal and neonatal mortality rates has been well demonstrated in a systematic review of studies conducted in similar settings in Sub-Saharan Africa [56]. Secondly, while our sample was systematic and sought to capture all or nearly all women who had delivered in the past year in the selected villages or hamlets, it was not a formal random sample. This limits the statistical generalizability of our findings. In addition, the insecurity at the time of the survey made it impossible to include the most remote payam. While we suspect that the situation is even worse in this remote area, we were unable to explore this hypothesis. Thirdly, although the women who participated in the community survey were interviewed about their most recent delivery (within the previous 12 months), some of them might not have correctly remembered some of the events that are reported in this study. Also because home health promoters accompanied the research assistants, the women could have reported the desired health behaviours more. While the decision to avoid audio-recording certain interviews was primarily informed by ethical considerations and guidelines to respect the preferences and comfort of respondents and may have increased the validity of the findings if respondents felt free to respond more openly, the lack of some audio-recordings may have limited the accuracy and completeness of the qualitative dataset, despite detailed fieldnotes taken by experienced qualitative researchers. Finally, because this study sought to explore a wide range of possible factors associated with where deliveries occur and because it was undertaken in the formative phase of an ongoing project, it was limited in the depth of exploration of important issues. In particular, the divergent perspectives of NGO and government staff on the one hand and women in communities on the other with respect to the importance of cultural preferences for home delivery merits further exploration, and the complex social and gender dynamics suggested by some of the findings are not fully examined in this study.

## Conclusion

This research has highlighted individual socio-demographic characteristics, economic barriers (ability to pay for care, in kind payments) and the perceived poor quality of care in the health facilities due to absence of health staff, lack of supplies and fear of discrimination based on social and economic status as the key barriers to institutional deliveries in Torit County, South Sudan. This study has shown the importance of understanding the interactions between the types of barriers influencing access to maternal health care services. Indeed, far from seeing evidence of “birth preparedness” in the conventional sense of planning and saving for an institutional delivery by a skilled birth attendant, our findings suggest that in this region of South Sudan women are actively “preparing” or at least planning for a home delivery attended by a TBA – in large part because the expectations of costly, poor quality and humiliating health facility care discourage women from seeking this care.

In terms of policy implications, interventions need to focus on the health system. Improving quality of care in both its social and technical dimensions is urgently needed to improve access to maternal health care services. Interventions need to be designed to tackle tacit or explicit demands for illegal unofficial payments and the fear of discrimination based on socio-economic status. Improving the provision of drugs and supplies and both technical and interpersonal capacity building of health providers are needed to improve access to maternal health care services. At the community level, interventions should focus on older pregnant or reproductive-aged women and relatives of pregnant women to promote deliveries at adequately equipped and staffed institutions. There is a particular need to improve equity in accessing maternal health services in rural areas like Himodonge Payam. Strategies to increase the coverage of ANC need to be maintained and enhanced as this seems to create the linkage for institutional deliveries and then PNC service utilization.

## Supplementary information

**Additional file 1.** Community survey Questionnaire.

## Data Availability

The datasets generated and/or analysed during the current study are available from the corresponding author on reasonable request.

## Abbreviations

ANC: Antenatal Care
CI: Confidence Interval
CRCHUM: Research center of Hospital Center of University of Montreal
DHIS: District Health Information System
EmOC: Emergency Obstetric Care
FGD: Focus Group Discussion
HPF: Health Pool Fund
LMIC: Low and Middle Income Countries
MCH: Maternal Child Health
MDGs: Millennium Developments Goals
MoH: Ministry of Health
NGOs: Non-Governmental Organizations
OR: Odds Ratio
PHCC: Primary Health Care Center
PHCU: Primary Health Care Unit
PNC: Post Natal Care
RMNCH: Reproductive Maternal Neonatal Child Health
SDGs: Sustainable Development Goals
SMoH: State Ministry of Health
SSHS: South Sudan Household Survey
STD: Standard Deviation
TBAs: Traditional Birth Attendants
UCL: University College of London
UHC: Universal Health Coverage

## Footnotes

1 The stamp refers to the receipt that is given to the patients when they pay for certain supplementary medical services

2 A bar of soap is about 60 South Sudanese pounds (ssp) ($ 1) and a packet of sweets is about 200 ssp. ($3.3)
