# Supplementary material for: “Midwives do not appreciate pregnant women who come to the maternity with torn and dirty clothing”: institutional delivery and postnatal care in Torit County, South Sudan: a mixed method study
Source: BMC Pregnancy Childbirth. 2020 Apr 28;20:250. doi: 10.1186/s12884-020-02910-2 (PMC7189725; doi:10.1186/s12884-020-02910-2)
Supplement: Supplementary file 1 — Additional file 1. Community survey Questionnaire. [file 12884_2020_2910_MOESM1_ESM.doc]

| **Questionnaire ID** | | | | |
| --- | --- | --- | --- | --- |
|  |  |  |  |  |

| **Mother ID** | | | | |
| --- | --- | --- | --- | --- |
|  |  |  |  |  |

#### **Community survey Questionnaire**

**MoCHeLaSS Project**

**Informed consent from the Mother:**

My name is ____________________ I work for MoCHeLaSS study, which is trying to improve the health status of mothers and babies in this area. We are carrying out a survey of women who have recently given birth, collecting information about their pregnancy, delivery and newborn baby. For this purpose I would like to talk to you in private. The interview will not take more than 1hour. I would like to assure you that the information collected through this interview will be kept confidential and used only for study purpose. You can decide freely whether you would like to take part in the interview. You can also refuse to answer any question that you do not like to answer. This survey is very important for improving the health status of mothers and babies. I would there fore appreciate your cooperation for the successful completion of the survey.

Interview procedure and process explained? O Yes O No

***If consent is given proceed with the interview, if consent is refused close the interview.***

**Section 0: general information**

| **No.** | **Question** | **Response** | **Code** | **Go to** |
| --- | --- | --- | --- | --- |
| 0.01 | Payam/ Sub-County | Kudu  Nyong  Himodonge  …………….  …………….  ……………. | 1  2  3  4  5  6 etc ( |  |
| 0.05 | Village name |  |  |  |
| 0.07 | Household identification note |  |  |  |
| 0.08 | Date of interview | _____/_____/____ (dd/mm/yy) |  |  |
| 0.09 | Name of interviewer |  |  |  |
| 0.10 | Name of quality controller |  |  |  |
| 0.11 | Initials of Mother |  |  |  |
| 0.12 | Name of newborn baby | No name | -88 |  |
| 0.13 | Name of provider or TBA interviewed *(If one was present at birth and mother or baby died)* | __________________________________________________________________ |  |  |
| 0.14 | Outcome for mother | Alive now  Died after childbirth  Died during labour / childbirth  Died during pregnancy | 1  2  3  4 |  |
| 0.15 | Outcome for baby | Alive now  Stillborn  Died in first month (within 28 days) | 1  2  3 |  |
| 0.16 | Initials of interviewee *(If mother has died)* | _________________________________ |  |  |

## SECTION 1: BACKGROUND CHARACTERISTICS

**First, I would like to ask you some questions about your background.**

| **No.** | **Question** | **Response** | **Code** | | **Go to** |
| --- | --- | --- | --- | --- | --- |
| 1.01 | How old were you at your last birthday? | _________Years  Don’t know | -99 | |  |
| 1.02 | What is your religion? | Catholic  Protestant  adventist  Islam  Other, please specify ................................ | 1  2  3  4  5 | |  |
|  |  |  |  | |  |
| 1.04 | Which ethnic groups do you belong to? | Lotuko  Acholi (ss)  Madi (ss)  Lango (ss)  Lokoya  Acholi (Ug)  Lango (Ug)  Madi (Ug)  Others……………………….. | 1  2  3  4  5  6  7  8 | | 1.07 |
| 1.05 | Have you ever attended school? If yes, what was the highest level of education achieved? | None  Primary  Secondary + | 1  2  3  4  5 | |  |
| 1.06 | Can you read this passage for me please? (*Interviewer to decide the ability level)* | Easily  With difficulty  Cannot read | 1  2  3 | |  |
|  |  |  | **Y** | **N** |  |
| 1.07 | Which of these do you presently have in your household? | 1. Electricity  2. Generator  3. Battery/Solar  4. Fan  5. TV  6. Fridge  7. Motorcycle  8. Radio  9.Bicycle  10. Radio | 1  1  1  1  1  1  1  1  1  1 | 2  2  2  2  2  2  2  2  2  2 |  |
| 1.08 | Do you have agricultural land? | Yes  No | 1  2 |  | 1.11  1.12 |
| 1.09 | If yes, how much land do you have? | Less than 2 acres  Between 2-4 acres  More than 4 acres  Land mortgaged | 1  2  3  4 |  |  |
| 1.10 | Do you use your money to pay healthcare for yourself? | Yes  No |  |  |  |
| 1.11 | Do you use your money to pay healthcare for your children | Yes  No |  |  |  |
| 1.12 | Do you have access to your husband’s money? | Yes  No  If No why? |  | |  |
| 1.13 | Can you freely use your resources without necessarily seeking for permission? | Yes  No, if no Why? |  | |  |
| 1.14 | With your own money or land, were you able to achieve activities such as? | - Starting income generating activities ( ie cooking, preparing local beer, traditional clothes, carpets..) Y/N - Buying things for yourself Y/ N - Buying things for the households (food, clothing, children education,) |  | |  |
| 1.15 | If you were ill, who would have the greatest say in the decision regarding to seek for healthcare? | Self  Mainly me  Me and my husband jointly  Mainly my husband  My husband  My mother in law  My father in law  Other, please specify ................................ | 1  2  3  4  5  6  7  8 | |  |
| 1.16 | If you were ill, would you go to a healthcare provider without the company of another adult? | Yes  No | 1  2  3  4 | |  |
| 1.16 | Whom do you usually go with to the market? | Alone  With a company of another adult | 1  2  3  4 | |  |
| 1.18 | Who has the greatest say in the decision regarding how household money is spent for daily necessities, such as food? | Self  Mainly me  Me and my husband jointly  Mainly my husband  My husband  My mother in law  My father in law  Other, please specify ................................ | 1  2  3  4  5  6  7  8 | |  |
| 1.19 | Who has the greatest say in the decision regarding how household money is spent for necessities purchased less frequently such as clothing, clothes for your children? | Self  Mainly me  Me and my husband jointly  Mainly my husband  My husband  My mother in law  My father in law  Other, please specify ................................ | 1  2  3  4  5  6  7  8 | |  |
| 1.20 | Who has the greatest say in the decision regarding how household money is spent for more expensive items such as bicycle, radio, fan, and furniture? | Self  Mainly me  Me and my husband  Mainly my husband  My husband  My mother in law  My father in law  Other, please specify ............................... | 1  2  3  4  5  6  7  8 | |  |
| 1.21 | How old were you when you first got married? | _________Years  Don’t know | -99 | |  |
| 1.22 | Who will pay for your healthcare? | Self  Husband  Father in law  Mother in law  Mother  Father  Other |  | |  |
| 1.23 | If your child were ill, who would have the greatest say in the decision to seek for healthcare? | Self  Husband  Father in law  Mother in law  Mother  Father  Other |  | |  |
| 1.24 | Who will pay for your child’s healthcare? | Self  Husband  Father in law  Mother in law  Mother  Father  Other |  | |  |
| 1.25 | How are women rewarded/ appreciated / recognized as a good woman in this society? | (open question) |  | |  |
| 1.26 | Is your work valued and appreciated? | Yes  No |  | |  |
| 1.27 | If yes, how is it rewarded/how do you know that it is valued and appreciated? | (open question) |  | |  |
| 1.28 | Who valued your work? | (open question) |  | |  |
| 1.29 | What do women do? | Chores (cleaning home, cooking)  Bringing woods  Bringing water  Working outside (generating outcome)  Taking care of children  Working the land  Breeding  Going to the market |  | |  |
| 1.30 | What do men do? | Chores  Bringing woods  Bringing woods  Working outside (generating outcome)  Taking care of children  Working the land  Breeding  Going to the market |  | |  |
| 1.20 | What was your age at first pregnancy? | _________Years  Don’t know | -99 | |  |
| 1.21 | How many times have you been pregnant in total, even if the pregnancy only lasted a short while or the baby subsequently died? | _________Times  Don’t know | -99 | |  |
| 1.22 | What was the date of birth for your most recent delivery?  *The one within the last six weeks* | _____/______/____ (dd/mm/yy)  Mother died during pregnancy | -88 | |  |
| 1.23 | How old was your previous child? | _____/______/____ (dd/mm/yy)  Only had one pregnancy  Don’t know | -88  -99 | |  |
| 1.24 | Have you heard of the women’s group in your area? | Yes  No  Name………………………………………  Don’t know | 1  2  -99 | |  |
| 1.25 | Do you currently attend any women’s group? If yes who is it organized by? | Do not attend any women’s group  Attend EIP women’s group  Attend another women’s groups please specify, ………………………………… | 1  2  3 | |  |

**SECTION 2: ANTENATAL CARE**

**Now I would like to ask you some questions about your most recent pregnancy.**

| **No.** | **Question** | **Response** | **Code** | | **Go to** |
| --- | --- | --- | --- | --- | --- |
| 2.01 | At the time you last became pregnant, did you want to become pregnant then, did you want to wait until later, or did you want no more children at all? | Wanted to become pregnant at that time  Wanted to wait until later  Did not want anymore children | 1  2  3 | |  |
| 2.02 | At the time you last became pregnant, did your husband want you to become pregnant then, did he want to wait until later, or did he want no more children at all? | Wanted to become pregnant at that time  Wanted to wait until later  Did not want anymore children | 1  2  3 | |  |
| 2.03 | Did you see a health care provider during your last pregnancy? | Yes  No | 1  2 | | 2.05  2.04 |
| 2.04 | If you did not see a health care provider during your last pregnancy, is there any particular reason why you did not?  *(Circle all that apply)* | Did not see a need to  Did not know where to go  Did not know about antenatal care  Provider is too far away  No transport to reach provider  Roads are bad  No one to accompany  Too busy to go to provider  Could not afford to go to provider  Family forbade to visit provider  No one able to look after children  Facility has inconvenient opening times  Nurse’s time of visit not known  Facility / provider has poor quality service  Did not like attitude of staff  No provider available at facility  Other, please specify………………………  ................................................................. | 1  2  3  4  5  6  7  8  9  10  11  12  13  14  15  16  17 | | 2.12 |
| 2.05 | If you did see a health care provider during your last pregnancy, who decided that you should? | Self  Husband  In-laws  Parents  NGO outreach worker  Neighbour  Women’s group member  Other, please specify ................................ | 1  2  3  4  5  6  7  8 | |  |
| 2.06 | What was the reason for your first visit to a health care provider? | Just for a check-up  For a problem  Don’t know | 1  2  -99 | |  |
| 2.07 | How many months pregnant were you when you first saw a health care provider? | .......... Month  Don’t know | -99 | |  |
| 2.08 | How many times in total did you see a health care provider during your last pregnancy? | ..............Times  Don’t know | -99 | |  |
| 2.09 | Where did you go for your **first** antenatal care visit? | PHCU  Primary health Care centre PHCC  District/State Hospital  Private facility  NGO facility  Charitable dispensary/hospital  Provider’s home  Other, please specify ................................ | 1  2  3  4  5  6  7  8 | |  |
| 2.10 | Whom did you see for antenatal care most of the time? | Doctor  Nurse/Midwife  Other outreach worker / NGO  TBA  Village doctor  Other, please specify ................................ | 1  2  3  4  5  6 | |  |
|  |  |  | **Y** | **N** |  |
| 2.11 | During any of your visits did the provider  (*Show picture card)* | 1.Measure your height  2.Weigh you  3.Examine your belly  4.Measure your blood pressure  5.Take a sample of your urine  6.Take some blood for tests  7.Perform a vaginal examination  8.Give information on breastfeeding  9.Give information on contraception  10.Give information on postnatal check  11.Give information on danger signs | 1  1  1  1  1  1  1  1  1  1  1 | 2  2  2  2  2  2  2  2  2  2  2 |  |
| 2.12 | Did you take iron tablets during your pregnancy? | Yes  No  Don’t know | 1  2  -99 | | 2.13  2.15  2.16 |
| 2.13 | If you took iron tablets during your last pregnancy, where did you get them from? | PHCU  Primary health centre  District/state Hospital  Other government hospital  Private facility  NGO facility  Charitable dispensary/hospital  Provider’s home  Medical Shop  Village Doctor  Friend/Relative  Other, please specify ................................ | 1  2  3  4  5  6  7  8  9  10  11  12 | |  |
| 2.14 | How long did you take them for? | < 1month  1-3 months  4-6 months  > 6 months | 1  2  3  4 | |  |
| 2.15 | Did you ever visit a government facility and ask for iron tablets when you were pregnant but the staff would not give you any? | Yes  No  Did not visit | 1  2  3 | |  |
| 2.16 | While you were pregnant were you given any injections to prevent your baby from getting tetanus after birth? | Yes  No | 1  2 | | 2.18  2.17 |
| 2.17 | If you did not receive any tetanus injections during pregnancy, did you have one in the last ten years? | Yes  No  Don’t know | 1  2  -99 | | 2.20 |
| 2.18 | If yes, where did you get your injection from? | The PHCU  Primary health centre-PHCC  District/State Hospital  Other government hospital  Private facility  NGO facility  Charitable dispensary/hospital  Provider’s home  Home visit  Medical shop  Village Doctor  Other, please specify ................................ | 1  2  3  4  5  6  7  8  9  10  11  12 | |  |
| 2.19 | How many tetanus injections did you receive during pregnancy? | .......... shots  Don’t know | -99 | |  |
| 2.20 | Did you ever visit a government facility and request a TT shot while you were pregnant but you were not given one? | Yes  No  Did not go | 1  2  3 | |  |
| 2.21 | How did the quantity of food you ate during pregnancy compare with what you ate before you were pregnant? | Ate less  Ate the same  Ate more | 1  2  3 | |  |
| 2.22 | Do you take alcoholic drinks? | Yes  No | 1  2 | | 2.23  2.24 |
| 2.23 | If yes, how did the quantity of handia you took during pregnancy compare with what you did before you were pregnant? | Stopped drinking  Drank less  Drank more  Same as before | 1  2  3  4 | |  |
| 2.24 | How much work did you do during pregnancy? | Same as before  Less than before  More than before | 1  2  3 | |  |
| 2.25 | How much of rest did you take during pregnancy? | Same as before  Less than before  More than before | 1  2  3 | |  |
| 2.26 | Did you suffer from any problems during pregnancy? | Yes  No | 1  2 | | 2.27  2.28 |
| 2.27 | If you did suffer from problems during pregnancy, what did you suffer from?  (Probe- *were these your only problems*? Also about malaria) | 1………………………………………………  2………………………………………………  3………………………………………………  4………………………………………………  5……………………………………………… |  | |  |
|  |  |  | **Y** | **N** |  |
| 2.28 | Did you suffer from any of the following problems during pregnancy?  *(Show picture card)* | 1.Swollen feet / legs / face  2.Looked pale  3.Felt weak / tired  4.Breathless when doing household tasks  5.Severe headache/Dizziness / fainting  6.Fits / seizures / convulsions / lost consciousness  7.Severe pain in stomach  8.Excessive vomiting  9.Fever for more than 1 day  10.Blurred vision / spots before eyes  11.Difficulty seeing at night  12.Foul smelling vaginal discharge  13.Pain or burning sensation while urinating  14.Vaginal bleeding  15.Jaundice  16.Reduced or no fetal movement  17.Malaria | 1  1  1  1  1  1  1  1  1  1  1  1  1  1  1  1  1  1 | 2  2  2  2  2  2  2  2  2  2  2  2  2  2  2  2  2  2 |  |
| **If no problems experienced in Questions 2.26, 2.27 or 2.28 move to 2.44** | | | | | |
| 2.29 | Did you, or someone on your behalf, decide to seek care for any problems you experienced during your pregnancy? | Yes  No | 1  2 | | 2.31  2.30 |
| 2.30 | If no, why did you, or someone on your behalf, decide not to seek care?  *(Tick all that apply)* | Did not see a need to  Did not know where to go  Did not know about antenatal care  Provider is too far away  No transport to reach provider  Roads are bad  No one to accompany  Too busy to go to provider  Could not afford to go to provider  Nurse’s time of visit not known  Family forbade to visit provider  No one able to look after children  Facility has inconvenient opening times  Facility / provider has poor quality service  Did not like attitude of staff  No provider available at facility  Other, please specify………………………  ................................................................. | 1  2  3  4  5  6  7  8  9  10  11  12  13  14  15  16  17 | | 2.44 |
| 2.31 | For which problem(s) did you decide to seek care for during your pregnancy? | 1………………………………………………  2………………………………………………  3………………………………………………  4………………………………………………  5……………………………………………… |  | |  |
| 2.32 | How long was it from the onset of illness until you, or someone on your behalf, decided to seek care? | Immediately  < 6 hours  6-12 hours  12-24 hours  > 24 hours, please specify…………..days  Don’t know | 1  2  3  4  5  -99 | |  |
| 2.33 | Where did you first seek care? | At home  PHCU  Primary health centre/PHCC  District/State Hospital  Private facility  NGO facility  Charitable dispensary/hospital  Provider’s home  Traditional/ Faith healers  TBA  Village Doctor  Other, please specify ................................ | 1  2  4  5  6  7  8  9  10  11  12  13 | | 2.34    2.35 |
| 2.34 | If you received care at home, how long was it from when you decided to seek care until you received care? | Immediately  < 6 hours  6-12 hours  12-24 hours  > 24 hours, please specify………….days  No care received  Don’t know | 1  2  3  4  5  6  -99 | | 2.38 |
| 2.35 | After you decided to seek care how long was it before you arrived at the health facility? | Immediately  < 6 hours  6-12 hours  12-24 hours  > 24 hours, please specify………….days  Don’t know | 1  2  3  4  5  -99 | |  |
| 2.36 | After arriving at the health facility how long was it before a member of staff provided care for you? | Immediately  < 6 hours  6-12 hours  12-24 hours  > 24 hours, please specify………….days  Don’t know  No care was received | 1  2  3  4  5  -99  -88 | | 2.38  2.37 |
| 2.37 | Why was no care received?  *(Circle all that apply)* | Facility closed  Facility had inadequate supplies / equipment  Staff too busy  Staff not available  Staff refused to see  Other, please specify………………………  ………………………………………………. | 1  2  3  4  5  6 | |  |
| 2.38 | Were you referred to another facility? | Yes  No | 1  2 | | 2.39  2.44 |
| 2.39 | What facility were you referred to? | Primary health centre/ PHC  District/State Hospital  Private facility  NGO facility  Charitable dispensary/hospital  Other, please specify ................................ | 1  2  3  4  5  6 | |  |
| 2.40 | Did you go there? | Yes  No | 1  2 | | 2.42  2.41 |
| 2.41 | Why didn’t you go there?  *(Circle all that apply)* | Did not see a need to  Did not know where to go  Did not know about antenatal care  Provider is too far away  No transport to reach provider  Roads are bad  Nurse ‘s visit not certain  No one to accompany  Too busy to go to provider  Could not afford to go to provider  Family forbade to visit provider  No one able to look after children  Facility has inconvenient opening times  Facility / provider has poor quality service  Did not like attitude of staff  No provider available at facility  Other, please specify………………………  ................................................................. | 1  2  3  4  5  6  7  8  9  10  11  12  13  14  15  16  17 | |  |
| 2.42 | Did you experience any difficulties while trying to access care? | Yes  No | 1  2 | | 2.43  2.44 |
| 2.43 | What difficulties did you experience while trying to access care?  *(Circle all that apply)* | Did not receive any treatment  Had to wait a long time for treatment  Facility had inadequate supplies / equipment  Did not see provider  Staff were not adequately qualified  Staff were rude  Transport problems to reach facility  Expensive / could not afford  Facility was closed  Other, please specify………………… | 1  2  3  4  5  6  7  8  9  10 | |  |
|  |  |  |  | |  |
| 2-44 | What kind of restrictions did you have during pregnancy? | Food  Alcohol  Work  Rest  Eclipse  Going to certain places  No restrictions  Any other… | 1  2  3  4  5  6  7  8 | |  |

**SECTION 3: DELIVERY**

**Now I would like to ask you a few questions about your most recent delivery *(one delivered in last six weeks)*.**

| **No.** | **Question** | **Response** | **Code** | | **Go to** |
| --- | --- | --- | --- | --- | --- |
| 3.01 | What was the duration of pregnancy when you delivered? | --------- months  Don’t know | -99 | |  |
| 3.02 | If you do not know the duration of pregnancy in months was the baby born … | Early  On time  Late | 1  2  3 | |  |
| 3.03 | Once the labour pains had become strong and regular, for how long did labour last? | < 12 hours  12-24 hours  > 24 hours |  | |  |
| 3.04 | Where was the baby delivered? | Government hospital  Charitable hospital  NGO facility  Private facility  Husband’s home  Parent’s home  Relative’s home  Other, please specify .............................. | 1  2  3  4  5  6  7  8 | | 3.05  3.06 |
| 3.05 | If the baby was delivered in a facility what was its name? | ……………………………………………………………………………………………… |  | |  |
| 3.06 | Did you have any known companion during delivery? | Yes  No | 1  2 | | 3.07  3.08 |
| 3.07 | If you had a known companion, who was it? | Mother in law  Mother  TBA  Husband  Other, please specify ................................ | 1  2  3  4  5 | |  |
| 3.09 | Who was the principal person who conducted the delivery? | Friend/Relative/ Neighbour  TBA  Doctor  Nurse  Husband  Other outreach worker  Village doctor  Self  Other, please specify ................................ | 1  2  3  4  5  6  7  8  9 | | 3.10  3.11  3.12 |
| 3.10 | If your baby was delivered by a friend or relative, approximately how many deliveries has she/he conducted in the last one year? | 1 - 2  3 - 5  6-10  >10  Don’t know | 1  2  3  4  -99 | | 3.12 |
| 3.12 | Was the person who conducted the delivery the person who you planned to conduct the delivery? | Yes  No  Made no plans | 1  2  3 | | 3.15  3.13  3.15 |
| 3.13 | If not, who did you plan to conduct the delivery? | Doctor  Nurse  Other outreach worker  Husband  TBA  Village doctor  Relative/Neighbor  Self  Other, please specify ................................ | 1  2  3  4  5  6  7  8  9 | |  |
| 3.14 | Why did you not have the person who you planned to conduct the delivery?  *(Circle all that apply)* | Planned person not available  Planned person refused to conduct  Not enough time to get to / fetch planned person  No tansport to get to / fetch planned person  No one available to take to / fetch planned person  Referred  Other, please specify……………………….  .................................................................. | 1  2  3  4  5  6  7 | |  |
| 3.15 | Who was the main decision-maker for your delivery? | Self  Me and my husband jointly  My husband  My mother in law  My father in law  Other, please specify ................................ | 1  2  3  4  5  6 | |  |
| 3.16 | Did you give anything either in cash or kind to the person who conducted the delivery? | Yes, paid cash and kind  Yes, paid in kind  Yes, paid in cash  No, gave nothing | 1  2  3  4 | | 3.17  3.17  3.18  3.19 |
| 3.17 | If you gave the person who conducted the delivery something in kind, what did you give? | Money  Food  Chicken/Goat  Soap  Other, please specify ................................ | 1  2  3  4  5 | |  |
| 3.18 | If you gave cash how much did you give, and if you paid in kind how much was the gift worth? | SSP/UGX ............. in Cash  Gift worth SSP/UGX……….. |  | |  |
| 3.19 | Did the person wash his / her hands with soap before assisting you? | Yes  No  Don’t know | 1  2  -99 | |  |
| 3.20 | Was a safe delivery kit used during the delivery?  *(Interviewer show safe delivery kit)* | Yes  No  Don’t know | 1  2  -99 | | 3.21  3.22  3.22 |
| 3.21 | Where did you get your safe delivery kit? | District/State Hospital  Primary Health Centre  NGO facility  Private Clinic  Medicine/Drug Shop  Other shop  TBA  Village Doctor  Self made  Other, please specify ..............................  Don’t know | 1  2  3  4  5  6  7  8  10  11  -99 | |  |
| 3.22 | Did you get any injections during the delivery? | Yes  No | 1  2 | | 3.23  3.25 |
| 3.26 | Was the baby delivered vaginally or was a caesarean section performed? If vaginal delivery, were instruments, such as a vacuum extractor or forceps used to help the baby out or did the baby arrive by itself? | Normal Vaginal  Vaginal with vacuum extractor  Vaginal with forceps  Caesarian Section | 1  2  3  4 | |  |
| 3.27 | What part of the baby came out first? | Head  Other, please specify……………………….  Don’t know | 1  2  -99 | |  |
| 3.28 | Did you suffer from any problems during delivery? | Yes  No | 1  2 | | 3.29  3.30 |
| 3.29 | If you did suffer from problems during delivery, what did you suffer from?  (*Probe-* *were these your only problems*?) | 1………………………………………………  2………………………………………………  3………………………………………………  4………………………………………………  5……………………………………………… |  | |  |
|  |  |  | **Y** | **N** |  |
| 3.30 | Did you suffer from any of the following during labour/delivery? | 1.High fever in the 3 days before labour  2.Foul smelling vaginal discharge in the 3 days before labour  3.Did the strong and regular labour pains last longer than 1 full day  4.Fits / seizures / convulsions / unconsciousness  5.Vaginal bleeding – so much that you thought you were going to die  6.Placenta still not out after half an hour / Needed medical help to get placenta out / someone had to put their hands inside your womb to get the afterbirth  7.Tear around the birth passage / vagina / cervix requiring stitching  8.Umbilical cord around babies neck | 1  1  1  1  1  1  1  1 | 2  2  2  2  2  2  2  2 |  |
| **If no problems experienced in Questions 3.28, 3.29or 3.30 move to SECTION 4** | | | | | |
| 3.31 | Did you, or someone on your behalf, decide to seek care for any problems you experienced during delivery? | Yes  No | 1  2 | | 3.33  3.32 |
| 3.32 | If no, why did you, or someone on your behalf, decide not to seek care?  *(Tick all that apply)* | Did not see a need to  Did not know where to go  Did not know about antenatal care  Provider is too far away  No transport to reach provider  Road too dangerous because of wild animals, elephants, etc.  No one to accompany  Too busy to go to provider  Could not afford to go to provider  Family forbade to visit provider  No one able to look after children  Facility has inconvenient opening times  Facility / provider has poor quality service  Did not like attitude of staff  No provider available at facility  Other, please specify………………………  ................................................................. | 1  2  3  4  5  6  7  8  9  10  11  12  13  14  15  16 | | Sec 4 |
| 3.33 | For which problem(s) did you decide to seek care for during your delivery? | 1……………………………………………  2……………………………………………  3……………………………………………  4……………………………………………  5…………………………………………… |  | |  |
| 3.34 | How long was it from the onset of illness until you, or someone on your behalf, decided to seek care? | Immediately  < 6 hours  6-12 hours  12-24 hours  > 24 hours, please specify …………..days  Don’t know | 1  2  3  4  5  -99 | |  |
| 3.35 | Where did you first seek care? | Home visit  District/State Hospital  PHC Centre  NGO facility  Private facility  At provider’s home  Other, please specify .............................  …………………………………………….. | 1  2  3  4  5  6  7 | | 3.36  3.37 |
| 3.36 | If you received care at home, how long was it from when you decided to seek care until you received care? | Immediately  < 6 hours  6-12 hours  12-24 hours  > 24 hours, please specify………….days  No care received  Don’t know | 1  2  3  4  5  6  -99 | | 3.40 |
| 3.37 | After you decided to seek care how long was it before you arrived at the health facility? | Immediately  < 6 hours  6-12 hours  12-24 hours  > 24 hours, please specify………….days  Don’t know | 1  2  3  4  5  -99 | |  |
| 3.38 | After arriving at the health facility how long was it before a member of staff provided care for you? | Immediately  < 6 hours  6-12 hours  12-24 hours  > 24 hours, please specify………….days  Don’t know  No care was received | 1  2  3  4  5  -99  -88 | | 3.40  3.39 |
| 3.39 | Why was no care received?  *(Circle all that apply)* | Facility closed  Facility had inadequate supplies / equipment  Staff too busy  Staff not available  Staff refused to see  Other, please specify……………………… | 1  2  3  4  5  6 | |  |
| 3.40 | Were you referred to another facility? | Yes  No | 1  2 | | 3.41  Sec.4 |
| 3.41 | What facility were you referred to? | Primary health centre  District Hospital  Charitable hospital  NGO facility  Private facility  Other, please specify ................................ | 1  2  3  4  5  6 | |  |
| 3.42 | Did you go there? | Yes  No | 1  2 | | 3.44  3.43 |
| 3.43 | Why didn’t you go there?  *(Circle all that apply)* | Did not see a need to  Did not know where to go  Did not know about antenatal care  Provider is too far away  No transport to reach provider  Roads are bad/fear of wild animals  No one to accompany  Too busy to go to provider  Could not afford to go to provider  Family forbade to visit provider  No one able to look after children  Facility has inconvenient opening times  Facility / provider has poor quality service  Did not like attitude of staff  No provider available at facility  Other, please specify……………………… | 1  2  3  4  5  6  7  8  9  10  11  12  13  14  15  16 | |  |
| 3.44 | Did you experience any difficulties while trying to access care? | Yes  No | 1  2 | | 3.45  Sec 4 |
| 3.45 | What difficulties did you experience while trying to access care?  *(Circle all that apply)* | Did not receive any treatment  Had to wait a long time for treatment  Facility had inadequate supplies / equipment  Did not see provider  Staff were not adequately qualified  Staff were rude  Transport problems to reach facility  Expensive / could not afford  Facility was closed  Other, please specify……………………… | 1  2  3  4  5  6  7  8  9  10 | |  |

**SECTION 4: POST-PARTUM**

**Now I would like to ask you a few questions about yourself since your most recent delivery.**

| **No.** | **Question** | **Response** | | **Code** | | **Go to** |
| --- | --- | --- | --- | --- | --- | --- |
| 4.01 | Did you receive a check-up, at home, or elsewhere, at any time during the six weeks after delivery? | Yes  No | | 1  2 | | 4.02  4.08 |
| 4.02 | How many days after delivery did you have your first check up? | ______ days  Don’t know | | -99 | |  |
| 4.03 | What was the reason for your first visit? | For a problem  Just for a check-up | | 1  2 | |  |
| 4.04 | How many times did you see a health care provider in the six weeks after delivery? | ______ times  Don’t know | | -99 | |  |
| 4.05 | Where did you have most of your check-ups? | PHCU/ HC II  Primary health centre/PHCC  District/State Hospital  Other government hospital  Private facility  NGO facility  Charitable dispensary/hospital  Provider’s home  Home visit  Village Doctor  Other, please specify ................................ | | 1  2  3  4  5  6  7  8  9  10  11 | |  |
| 4.06 | Who did you see for your post-natal check-ups most of the time? | Doctor/health assistant/paramedic  Nurse/Midwife  Other outreach worker  TBA  Village doctor  Other, please specify ................................ | | 1  2  3  4  5  6 | |  |
|  |  |  | | **Y** | **N** |  |
| 4.07 | During any of your check-ups did the provider,,,  *(Show picture cards)* | 1.Measure your blood pressure 2.Temperature  3.Test your Blood  4.Examine your belly  5.Breast examination  6.Vaginal examination  7.Advice on breastfeeding  8.Advice on nutrition for mother  9.Advice on family planning / birth spacing  10.Advice on baby care  11.Advice on danger signs | | 1  1  1  1  1  1  1  1  1  1  1 | 2  2  2  2  2  2  2  2  2  2  2 |  |
| 4.08 | Did you suffer from any problems since delivery? | Yes  No | | 1  2 | | 4.09  4.10 |
| 4.09 | If you suffered from problems after delivery, what problems did you suffer from? | 1………………………………………………2………………………………………………3………………………………………………4………………………………………………5………………………………………….….. | |  | |  |
|  |  |  | | **Y** | **N** |  |
| 4.10 | Did you suffer from any of the following after delivery? | 1.Pale  2.Weak / tired  3.Breathlessness when doing household tasks  4.Dizziness / fainting  5.Fits / seizures / convulsions / unconsciousness  6.Severe pain in stomach  7.Fever for more than 24 hours  8.Foul smelling vaginal discharge  9.Vaginal bleeding – so much that you thought you were going to die  10.Leaking of urine or stool from vagina  11.A feeling of heaviness like your womb or vaginal wall is falling out, or did you actually see or feel it externally  12. Feel the womb coming out | | 1  1  1  1  1  1  1  1  1  1  1  1 | 2  2  2  2  2  2  2  2  2  2  2  2 |  |
| **If no problems experienced in Questions 4.08, 4.09 or 4.10 move to 4.26** | | | | | | |
| 4.11 | Did you, or someone on your behalf, decide to seek care for any problems you experienced after delivery? | Yes  No | | 1  2 | | 4.13  4.12 |
| 4.12 | If no, why did you, or someone on your behalf, decide not to seek care?  *(Circle all that apply)* | Did not see a need to  Did not know where to go  Did not know about postnatal care  Provider is too far away  No transport to reach provider  Roads were bad  No one to accompany  Too busy to go to provider  Could not afford to go to provider  Family forbade to visit provider  No one able to look after children  Facility has inconvenient opening times  Facility / provider has poor quality service  Did not like attitude of staff  No provider available at facility  Other, please specify………………………  ................................................................. | | 1  2  3  4  5  6  7  8  9  10  11  12  13  14  15  16 | | 4.26 |
| 4.13 | For which problem(s) did you decide to seek care for after your delivery? | 1……………………………………………  2……………………………………………  3……………………………………………  4……………………………………………  5…………………………………………… | |  | |  |
| 4.14 | How long was it from the onset of illness until you, or someone on your behalf, decided to seek care? | Immediately  < 6 hours  6-12 hours  12-24 hours  > 24 hours, please specify…………..days  Don’t know | | 1  2  3  4  5  -99 | |  |
| 4.15 | Where did you first seek care? | Home visit  PHCU/HC II  Primary Health Centre/PHCC  District/State Hospital  NGO facility  Private facility  At provider’s home  Other, please specify ................................ | | 1  2  3  4  5  6  7  8  9 | | 4.16  4.17 |
| 4.16 | If you received care at home, how long was it from when you decided to seek care until you received care? | Immediately  < 6 hours  6-12 hours  12-24 hours  > 24 hours, please specify………….days  No care received  Don’t know | | 1  2  3  4  5  6  -99 | | 4.20 |
| 4.17 | After you decided to seek care how long was it before you arrived at the health facility? | Immediately  < 6 hours  6-12 hours  12-24 hours  > 24 hours, please specify………….days  Don’t know | | 1  2  3  4  5  -99 | |  |
| 4.18 | After arriving at the health facility how long was it before a member of staff provided care for you? | Immediately  < 6 hours  6-12 hours  12-24 hours  > 24 hours, please specify………….days  Don’t know  No care was received | | 1  2  3  4  5  -99  -88 | | 4.20  4.19 |
| 4.19 | Why was no care received?  *(Circle all that apply)* | Facility closed  Facility had inadequate supplies / equipment  Staff too busy  Staff not available  Staff refused to see  Other, please specify………………………  ………………………………………………. | | 1  2  3  4  5  6 | |  |
| 4.20 | Were you referred to another facility? | Yes  No | | 1  2 | | 4.21  4.24 |
| 4.21 | What facility were you referred to? | Primary health centre/PHCC  District/State Hospital  Charitable hospital  NGO facility  Private facility  Other, please specify ................................ | | 1  2  3  4  5  6 | |  |
| 4.22 | Did you go there? | Yes  No | | 1  2 | | 4.24  4.23 |
| 4.23 | Why didn’t you go there?  *(Circle all that apply)* | Did not see a need to  Did not know where to go  Did not know about postnatal care  Provider is too far away  No transport to reach provider  Roads were bad  No one to accompany  Too busy to go to provider  Could not afford to go to provider  Family forbade to visit provider  No one able to look after children  Facility has inconvenient opening times  Facility / provider has poor quality service  Did not like attitude of staff  No provider available at facility  Other, please specify………………………  ................................................................. | | 1  2  3  4  5  6  7  8  9  10  11  12  13  14  15  16 | |  |
| 4.24 | Did you experience any difficulties while trying to access care? | Yes  No | | 1  2 | | 4.25  4.26 |
| 4.25 | What difficulties did you experience while trying to access care?  *(Circle all that apply)* | Did not receive any treatment  Had to wait a long time for treatment  Facility had inadequate supplies / equipment  Did not see provider  Staff were not adequately qualified  Staff were rude  Transport problems to reach facility  Expensive / could not afford  Facility was closed  Other, please specify………………………  ………………………………………………. | | 1  2  3  4  5  6  7  8  9  10 | |  |
| 4.26 | What were the restrictions during the postpartum period? | Kept in separate room (untouchable)  Not allowed to bathe  Allowed only one meal a day  Food restrictions  No restrictions  Any other, specify…. | 1  2  3  4  5  6 | -days  -days  -days  -days  -days  -days | |  |

**SECTION 5: THE NEWBORN**

**Now I would like to ask you some questions specifically relating to your last baby.**

***If the mother had a multiple pregnancy please ensure that you complete Section 5 separately for each baby.***

| **No.** | **Question** | **Response** | **Code** | | | **Go to** |
| --- | --- | --- | --- | --- | --- | --- |
| 5.01 | Did you have a multiple pregnancy? | Yes  No | 1  2 | | | 5.02  5.03 |
| 5.02 | If a multiple pregnancy, was this baby born … | First  Second  Don’t know | 1  2  -99 | | |  |
| **Please complete the following questions for each child** | | | | | | |
| 5.03 | Is the baby male or female? | Male  Female | 1  2 | |  | |
| 5.04 | What was the baby’s size at birth? | Smaller than average size  Normal size  Larger than average size | 1  2  3 | |  | |
| 5.05 | Did the baby look normal or abnormal at birth? | Normal  Abnormal  Don’t know | 1  2  -99 | | 5.07  5.06  5.07 | |
| 5.06 | In what way do you think the baby looked abnormal? | ……………………………………………...………………………………………………...……… |  | |  | |
| 5.07 | Did the baby have bruises or signs of injury? | Yes  No | 1  2 | | 5.13 | |
| 5.08 | When your baby was born did anyone do something to help the baby breathe? | Yes  No  Don’t know | 1  2  -99 | | 5.09  5.11  5.11 | |
| 5.09 | Who did something to help the baby breathe? | Doctor  Nurse  TBA  Village doctor  Husband  Relative/Friend  Other, please specify .............................. | 1  2  3  4  5  6  7 | |  | |
| 5.10 | What did they do?  *(Circle all that apply)* | Held baby upside down  Smacked bottom  Flicked feet  Blew into the ears  Put cold water on the baby  Threw the baby up in the air  Mouth to mouth  Tube and mask  Bag and mask  Intubated  Other, please specify…………………….. | 1  2  3  4  5  6  7  8  9  10  11 | |  | |
| 5.11 | Did your baby breathe or cry IMMEDIATELY after birth? | Yes  No | 1  2 | | 5.12  6.01 | |
| 5.12 | Did the baby ever cry, move or breathe, even if it was only for a very short time? | Yes  No  Don’t know | 1  2  -99 | | 5.13  6.01  6.01 | |
| 5.13 | When did the baby first breathe or cry? | Very soon after birth (within 1 minute)  Soon after birth (1-5 minutes)  A long time after birth (5-30 minutes) | 1  2  3 | |  | |
| 5.14 | 5 minutes after birth which of these descriptions best describes your baby? | Crying well, breathing well,  Pink and active  Poor cry, poor breathing, blue limbs,  Poorly active  No cry, blue body, slow heartbeat, no movement  Don’t know | 1  2  3  4  5  -99 | |  | |
| 5.17 | How long after the baby’s birth did the placenta come out? | Within 30 minutes  30 minutes to 1 hour  1 hour to 6 hours  More than 6 hours  Don’t know | 1  2  3  4  -99 | |  | |
| 5.19 | What was the cord cut with after delivery? | New Blade  Old Blade  Knife  Scissor  Other, please specify ................................  Don’t know | 1  2  3  4  5  -99 | |  | |
| 5.20 | Was the instrument bought specifically for cutting the cord and unused? | Yes  No  Don’t know | 1  2  -99 | |  | |
| 5.21 | Was the instrument boiled prior to cutting the cord? | Yes  No  Don’t know | 1  2  -99 | |  | |
| 5.22 | Was the string used to tie the cord boiled prior to using? | Yes  No  Don’t know | 1  2  -99 | |  | |
| 5.23 | What was put on the cord after it was cut? | Nothing  Oil  Antiseptic  Mud  Talcom powder  Turmeric  Cloth  Animal dung  Ash / soot  Other, please specify ............................ | 1  2  3  4  5  6  7  8  9  10  11 | |  | |
| 5.24 | Birth weight | ----------Kg /  Weight was not taken  Don’t know | -88  -99 | |  | |
| 5.25 | How long after birth was the baby wiped? | Baby was not wiped  Immediately (< 10 minutes)  10-30 minutes  30 minutes – 1 hour  1-4 hours  > 4 hours  Don’t know | 1  2  3  4  5  6  -99 | |  | |
| 5.26 | How long after birth was the baby wrapped? | Baby was not wrapped  Immediately (< 10 minutes)  10-30 minutes  30 minutes – 1 hour  1-4 hours  > 4 hours  Don’t know | 1  2  3  4  5  6  -99 | | 5.28  5.27 | |
| 5.27 | Was the baby wrapped with a washed or unwashed cloth? | Washed / Clean  Unwashed  Don’t know | 1  2  -99 | |  | |
| 5.28 | How long after birth was the baby bathed? | Baby was not bathed  Immediately  Within 6 hours  7-24 hours  > 24hours  Don’t know | 1  2  3  4  5  -99 | |  | |
| 5.29 | How soon was the baby placed on the mother’s skin? | Baby was not placed on mother’s skin  Immediately (< 10 minutes)  10-30 minutes  30 minutes – 1 hour  1-4 hours  > 4 hours  Don’t know | 1  2  3  4  5  6  -99 | |  | |
| 5.30 | What was the first food ever given to your baby? | Breast milk  Other, please specify .............................. | 1  2 | |  | |
| 5.31 | When did you first put your baby to the breast? | Baby was never put to the breast  Immediately (< 10 minutes)  10-30 minutes  30 minutes – 1 hour  1-4 hours  4-24 hours  > 24 hours  Don’t know | 1  2  3  4  5  6  7  -99 | |  | |
| 5.32 | When you placed your baby to the breast did it ever have difficulty feeding on the first day? | Baby had difficulty on first day  Baby had no difficulty on first day  Baby was never put to breast on first day | 1  2  3 | | 5.33  5.34  5.34 | |
| 5.33 | If the baby had difficulty feeding on the first day, after how many days was the baby able to feed by itself? | ________days  Don’t know | -99 | |  | |
| 5.34 | Have you given the baby anything other than breast milk since birth? | Yes  No | 1  2 | | 5.35  5.37 | |
| 5.35 | What have you given the baby to eat or drink? | ______________________________________________________________________ |  | |  | |
| 5.36 | How old was the baby the first time you gave the baby something other than breast milk? | 1 day  2-7 days  1-3 weeks  4-6 weeks  Don’t know | 1  2  3  4  -99 | |  | |
| 5.37 | Has your baby had the BCG injection? | Yes  No | 1  2 | |  | |
| 5.38 | Have you been for a check-up for your baby since delivery (within 0-28 days)? | Yes  No | 1  2 | | 5.39  5.44 | |
| 5.39 | How many times? | ______ times  Don’t know | -99 | |  | |
| 5.40 | How many days after delivery did your baby have the 1^st^ check up? | ______ days  Don’t know | -99 | |  | |
| 5.41 | Where did you go? | PHCU  Primary health centre/PHCC  District/State Hospital  NGO facility  Private facility  Provider’s home  Home visit  Other, please specify .............................. | 1  2  3  4  5  6  7  8 | |  | |
| 5.42 | Who did you see? | Doctor  Nurse/Other outreach worker  TBA  Village doctor  Relative / friend  Other, please specify .............................. | 1  2  3  4  5  6 | |  | |
| 5.43 | What was the reason for your first visit? | Baby had a problem  Just for a check-up | 1  2 | |  | |
| 5.44 | Is the baby alive now? | Yes  No | 1  2 | | 5.45  7.01 | |
| 5.45 | Has the baby suffered from any problems during the first 28 days after birth? | Yes  No | 1  2 | | 5.46  5.47 | |
| 5.46 | What problems did the baby suffer from? | ____________________________________________________________________________________________________________________________________________ |  | |  | |
|  |  |  | **Y** | **N** |  | |
| 5.47 | Did the baby suffer from any of the following problems … | 1- Born too early  2- Born too small  3- Didn’t cry properly / poor cry  4- Continuous crying  5- Didn’t breath properly  6- Squashed head when born  7- Vomiting repeatedly  8- Fits / seizures / convulsions / lost consciousness  9- Cold  10- Blue in colour  11- Infected umbilical cord  12- Fever for more than 24hrs  13- Yellow on first day of life  14- Yellow after first day of life  15- Cough  16- Diarrhoea more than 3 times day | 1  1  1  1  1  1  1  1  1  1  1  1  1  1  1  1 | 2  2  2  2  2  2  2  2  2  2  2  2  2  2  2  2 |  | |
| **If no problems experienced in Questions 5.45, 5.46or 5.47,then END** | | | | | | |
| 5.48 | Did you, or someone on your behalf, decide to seek care for any problems you experienced for your baby? | Yes  No | 1  2 | | | 5.50  5.49 |
| 5.49 | If no, why did you, or someone on your behalf, decide not to seek care?  *(Circle all that apply)* | Did not see a need to  Did not know where to go  Provider is too far away  No transport to reach provider  Roads were bad  No one to accompany  Too busy to go to provider  Could not afford to go to provider  Family forbade to visit provider  No one able to look after children  Facility has inconvenient opening times  Facility / provider has poor quality service  Did not like attitude of staff  No provider available at facility  Other, specify………………………................ | 1  2  3  4  5  6  7  8  9  10  11  12  13  14  15 | | | END |
| 5.50 | For which problem(s) did you decide to seek care for your baby? | 1……………………………………………  2……………………………………………  3……………………………………………  4……………………………………………  5…………………………………………… |  | | |  |
| 5.51 | How long was it from the onset of illness until you, or someone on your behalf, decided to seek care? | Immediately  < 6 hours  6-12 hours  12-24 hours  > 24 hours, please specify…………..days  Don’t know | 1  2  3  4  5  -99 | | |  |
| 5.52 | Where did you first seek care? | At home  PHCU/HC II  Primary health centre/PHCC  District/State Hospital  Charitable hospital  NGO facility  Private facility  At provider’s home  Other, please specify ............................... | 1  2  3  4  5  6  7  8  9 | | | 5.53  5.54 |
| 5.53 | If you received care at home, how long was it from when you decided to seek care until you received care? | Immediately  < 6 hours  6-12 hours  12-24 hours  > 24 hours, please specify………….days  No care received  Don’t know | 1  2  3  4  5  -88  -99 | | | 5.57 |
| 5.54 | After you decided to seek care how long was it before you arrived at the health facility? | Immediately  < 6 hours  6-12 hours  12-24 hours  > 24 hours, please specify………….days  Don’t know | 1  2  3  4  5  -99 | | |  |
| 5.55 | After arriving at the health facility how long was it before a member of staff provided care for you? | Immediately  < 6 hours  6-12 hours  12-24 hours  > 24 hours, please specify………….days  Don’t know  No care was received | 1  2  3  4  5  -99  -88 | | | 5.57  5.56 |
| 5.56 | Why was no care received?  *(Circle all that apply)* | Facility closed  Facility had inadequate supplies / equipment  Staff too busy  Staff not available  Staff refused to see  Other, please specify………………………. | 1  2  3  4  5  6 | | |  |
| 5.57 | Were you referred to another facility? | Yes  No | 1  2 | | | 5.58  5.61 |
| 5.58 | What facility were you referred to? | District/state Hospital  Charitable hospital  NGO facility  Private facility  Other, please specify ................................ | 1  2  3  4  5 | | |  |
| 5.59 | Did you go there? | Yes  No | 1  2 | | | 5.61  5.60 |
| 5.60 | Why didn’t you go there?  *(Circle all that apply)* | Did not see a need to  Did not know where to go  Did not know about antenatal care  Provider is too far away  No transport to reach provider  Roads were bad  No one to accompany  Too busy to go to provider  Could not afford to go to provider  Family forbade to visit provider  No one able to look after children  Facility has inconvenient opening times  Facility / provider has poor quality service  Did not like attitude of staff  No provider available at facility  Other, please specify………………………  ................................................................. | 1  2  3  4  5  6  7  8  9  10  11  12  13  14  15  16 | | |  |
| 5.61 | Did you experience any difficulties while trying to access care? | Yes  No | 1  2 | | | 5.62  END |
| 5.62 | What difficulties did you experience while trying to access care?  *(Circle all that apply)* | Did not receive any treatment  Had to wait a long time for treatment  Facility had inadequate supplies / equipment  Did not see provider  Staff were not adequately qualified  Staff were rude  Transport problems to reach facility  Expensive / could not afford  Facility was closed  Other, please specify………………………  ………………………………………………. | 1  2  3  4  5  6  7  8  9  10 | | |  |

***Section 6 Gender based violence, effect of war and disability***

***Thank you very much for helping us to learn in detail about your experiences with pregnancy and childbirth and your baby’s health. Now we would like to ask a few questions about how men and women relate to each other and your thoughts about the war and how it has affected your life***

| 6 | Questions | Response | Code |  |
| --- | --- | --- | --- | --- |
| 6.1 | Do you know a woman who has been abused by her husband/ partner? | Yes  No |  |  |
| 6.2 | If yes, What kind of abuses she was suffering of? | - Physical - Emotional - Sexual |  |  |
|  | Have you been abused by your husband/ partner? | - Yes - No |  |  |
|  | If yes, what kind of abused are you suffered from? | - Physical - Emotional - Sexual |  |  |
| 6.3 | During the war, have you been displaced? | - Yes - No |  |  |
| 6.4 | If yes, for how long? |  |  |  |
| 6.5 | During the war, have you been kidnapped? | - Yes - No |  |  |
| 6.6 | During the war, was a member of your family kidnapped? | - Yes - No |  |  |
| 6.7 | During the war, did someone from your family die? | - Yes - No |  |  |
| 6.8 | If yes, how are you related to that person? | - Husband - Children - Brother - Sister - Mother - Father - Uncle |  |  |
| 6.9 | During the war, did one of your relative was seriously/permanently injured? | - Yes - No |  |  |
| 6.10 | If yes, who | - Husband - Children - Mother - Father - Brother - Sister - Uncle |  |  |

***Thank you very much for your co-operation.***

| **Baby ID** | | | | |
| --- | --- | --- | --- | --- |
|  |  |  |  |  |

| **Mother ID** | | | | |
| --- | --- | --- | --- | --- |
|  |  |  |  |  |

**SECTION 6: STILLBIRTH**

**Relative / Provider *Delete as applicable***

**I would like to ask you some questions to try to help me understand exactly how and why the baby died. I understand that this is difficult for you to talk about and I am very grateful for any information that you are able to provide.**

**Please circle who was interviewed:**

1. Mother

2. Provider

3. Other, please specify relationship to child ___________________________________________

Name___________________________________________________Date___________________

| **No.** | **Question** | **Response** | | | **Code** | **Go to** |
| --- | --- | --- | --- | --- | --- | --- |
| 6.01 | Was the baby moving when you went into labour? | Yes  No  Don’t know | | | 1  2  -99 |  |
| 6.02 | When did you last feel the baby moving? | -----------hours before delivery  -----------days before delivery  Don’t know | | | -99 |  |
| 6.03 | Do you think the baby died before you went into labour? | Yes  No  Don’t know | | | 1  2  -99 |  |
| 6.04 | When you looked at the baby, did the body skin color look like the body of a normal baby or had it changed in some way? | Yes  No  Don’t know | | | 1  2  -99 |  |
| 6.05 | Did the baby have a very small head?(show photo of anencephaly) | Yes  No | | | 1  2 |  |
| 6.06 | Did the baby have a mass or defect on the back of the head or spine? (show photo of meningomyelocele) | Yes  No | | | 1  2 |  |
| 6.07 | Did the baby have a cleft lip or palate? (show photo of cleft lip and palate) | Yes  No | | | 1  2 |  |
| 6.08 | Did the baby have abnormal arms or legs? | Yes  No | | | 1  2 |  |
| 6.09 | I would really appreciate it if you could help me to try to understand exactly how and why the baby died. Please tell me everything that happened even if it means repeating some of the information that you have already provided.  ____________________________________________________________________________________________________________________________________________________________________________________________________________________________________________________________________________________________________________________________________________________________________________________________________________________________________________________________________________________________________________________________________________________________________________________________________________________________________________________________________________________________________________________________________________________________________  ________________________________________________________________________________________________________________________________________________________________________________________________________________________________________________________________________________________________ | | | | | |
| 6.10 | Do you think the stillbirth could have been prevented? | | Yes  No  Don’t know | 1  2  99 | | END  END |
| 6.11 | How do you think it could have been prevented?  ______________________________________________________________________________________________________________________________________________________________________________________________________________________________  ______________________________________________________________________________________________________________________________________________________________________________________________________________________________ | | | | | |

| **Baby ID** | | | | |
| --- | --- | --- | --- | --- |
|  |  |  |  |  |

| **Mother ID** | | | | |
| --- | --- | --- | --- | --- |
|  |  |  |  |  |

**SECTION 7: NEONATAL VERBAL AUTOPSY (Mother / Relative)**

**I would like to ask you some questions to try to help me understand exactly how and why the baby died. I understand that this is difficult for you to talk about and I am very grateful for any information that you are able to provide.**

Initials___________________________________________ Relationship to baby_______________

Date______________________

| **No.** | **Question** | **Response** | **Code** | **Go to** |
| --- | --- | --- | --- | --- |
| 7.01 | Initials of the baby |  |  |  |
| 7.02 | Date of birth of baby | ……./…../….. (dd/mm/yy) |  |  |
| 7.03 | Date of death of baby | ……./…../….. (dd/mm/yy) |  |  |
| 7.04 | I would really appreciate it if you could help me to try to understand exactly how and why the baby died. Please tell me everything that happened even if it means repeating some of the information that you have already provided.  ____________________________________________________________________________________________________________________________________________________________________________________________________________________________________________________________________________________________________________________________________________________________________________________________________________________________________________________________________________________________________________________________________________________________________________________________________________________________________________________________________________________________________________________________________________________________________  __________________________________________________________________________ | | | |
| 7.05 | Where did the baby die | Home  On the way to treatment  Health facility  Traditional healer’s place  Other, please specify …….. | 1  2  3  4  5 |  |
| 7.06 | Did the baby have a very small head?(show photo of anencephaly) | Yes  No | 1  2 |  |
| 7.07 | Did the baby have a mass or defect on the back of the head or spine? (show photo of meningomyelocele) | Yes  No | 1  2 |  |
| 7.08 | Did the baby have a cleft lip or palate? (show photo of cleft lip and palate) | Yes  No | 1  2 |  |
| 7.09 | Did the baby have abnormal arms or legs? | Yes  No | 1  2 |  |
| 7.10 | Did the baby have bruises or signs of injury? | Yes  No  Don’t know | 1  2  -99 |  |
| 7.11 | Was the baby sleepy and floppy at the time of birth? | Yes  No | 1  2 |  |
| 7.12 | Did the baby ever have difficulty breathing? | Yes  No | 1  2 | 7.13  7.21 |
| 7.13 | If the baby had difficulty breathing, what was the difficulty? (demonstrate) | Intermittent  Fast breathing | 1  2 |  |
| 7.14 | If the baby had difficulty breathing, when did the difficulty start? | Immediately after birth  Not immediately, but within 6 hours  More than 6 hours after birth | 1  2  3 |  |
|  |  |  |  |  |
| 7.15 | If the baby had difficulty breathing, how long did the difficulty continue? | _____________ days |  |  |
| 7.16 | If the baby had difficulty breathing, did it continue till the baby died? | Yes  No | 1  2 |  |
| 7.17 | If the baby had difficulty breathing, was there chest indrawing?  (demonstrate) | Yes  No | 1  2 |  |
| 7.18 | If the baby had difficulty breathing, did it grunt?(demonstrate) | Yes  No | 1  2 |  |
| 7.19 | If the baby had difficulty breathing, did it flare its nostrils? (demonstrate) | Yes  No | 1  2 |  |
| 7.20 | If the baby had difficulty breathing, did the baby have pneumonia? | Yes  No | 1  2 |  |
| 7.21 | Did the baby ever suckle normally? | Yes  No | 1  2 | 7.22  7.23 |
| 7.22 | Did the baby always suckle normally? | Yes  No | 1  2 | 7.29  7.23 |
| 7.23 | If the baby did not always suckle normally, when did the problem start? | On the 1^st^ day  After the 1^st^ day but in the 1^st^ 3 days  After the 1^st^ 3 days | 1  2  3 |  |
| 7.24 | If the baby did not always suckle normally, for how long did the problem continue? | ______________days |  |  |
| 7.25 | If the baby did not always suckle normally, did the feeding problem continue until the baby died? | Yes  No | 1  2 |  |
| 7.26 | If the baby did not always suckle normally, could the baby open his/her mouth? | Yes  No | 1  2 |  |
| 7.27 | If the baby did not always suckle normally, did the baby arch his/her back and have spasms?(show picture) | Yes  No | 1  2 |  |
| 7.28 | If the baby did not always suckle normally, did the baby have tetanus? | Yes  No | 1  2 |  |
| 7.29 | Was the baby’s stool more liquid than normal? | Yes  No | 1  2 |  |
| 7.30 | Did the baby pass stool more frequently than normal? | Yes  No | 1  2 |  |
| 7.31 | If the baby had diarrhoea, was there mucus or blood in the stool? | Yes  No | 1  2 |  |
| 7.32 | If the baby had diarrhoea, how long did it continue? | _______________days |  |  |
| 7.33 | Did the baby vomit everything? | Yes  No | 1  2 |  |
| 7.34 | Did the baby have fever? | Yes  No | 1  2 | 7.35  7.37 |
| 7.35 | If the baby had fever, how many days after birth did the fever start? | _____________days |  |  |
|  |  |  |  |  |
| 7.36 | If the baby had fever, for how long did the fever continue? | ________________days |  |  |
| 7.37 | Were the baby’s skin and eyes very yellow? | Yes  No | 1  2 |  |
| 7.38 | Did the baby have any seizures/ fits/convulsions for the first time? | Yes  No | 1  2 | 7.39  7.40 |
| 7.39 | On which day of life did the baby have fits/seizures? | First day  After first day |  |  |
| 7.40 | Did the baby feel cold? | Yes  No | 1  2 | 7.41  7.43 |
| 7.41 | If the baby felt cold, at how many days after birth did the baby start feeling cold? | _______________days |  |  |
| 7.42 | If the baby felt cold, for how long did the baby feel cold? | ______________Hours  ______________Days |  |  |
| 7.43 | Did the baby have pustules on the skin? | Yes  No | 1  2 |  |
| 7.44 | Did the baby have ear discharge? | Yes  No | 1  2 |  |
| 7.45 | Did the baby have red eyes with pus in them? | Yes  No | 1  2 |  |
| 7.46 | Did the baby have a bright red ring on the skin around the umbilicus cord stump? | Yes  No | 1  2 |  |
| 7.47 | Did the baby bleed? | Yes  No | 1  2 | 7.48  7.49 |
| 7.48 | From where did the baby bleed? | ___________________________________________________________________________ |  |  |
| 7.49 | What did the baby’s fontanelle look like?(point to fontanelle) | Sunken down  Normal  Bulging up |  |  |
| 7.50 | Did the baby become drowsy and unconscious when it had been normal before? | Yes  No | 1  2 |  |
| 7.51 | How long was the baby ill before he/she died? | ____________days |  |  |
| 7.52 | Did the baby die without any sign of illness? | Yes  No | 1  2 |  |
| 7.53 | Did the baby have some other problem that we have not discussed? | Yes  No | 1  2 | 7.54  7.55 |
| 7.54 | If Yes, what problem? | ___________________________________________________________________________ |  |  |
| 7.55 | Do you think the death could have been prevented? | Yes  No  Don’t know | 1  2  -99 | 7.56  END  END |
| 7.56 | How do you think the death could have been prevented? | _________________________  __________________________________________________________________________ |  |  |

***Thank you very much for your cooperation.***

| **Baby ID** | | | | |
| --- | --- | --- | --- | --- |
|  |  |  |  |  |

| **Mother ID** | | | | |
| --- | --- | --- | --- | --- |
|  |  |  |  |  |

**SECTION 8: NEONATAL VERBAL AUTOPSY (Provider)**

**I would like to ask you some questions to try to help me understand exactly how and why the baby died.**

Initials of interviewee___________________________ Position________________________

Date_____________________

**N.B. For provider check answer to Q 5.12, if not complete, Section 6, if complete, Section 8**

| 8.01 | Name of the baby |  |  |  |
| --- | --- | --- | --- | --- |
| 8.02 | Date of birth of baby | ……./…../….. (dd/mm/yy) |  |  |
| 8.03 | Date of death of baby | ……./…../….. (dd/mm/yy) |  |  |
| 8.04 | I would really appreciate it if you could help me to try to understand exactly how and why the baby died. Please tell me everything that happened even if it means repeating some of the information that you have already provided.  ____________________________________________________________________________________________________________________________________________________________________________________________________________________________________________________________________________________________________________________________________________________________________________________________________________________________________________________________________________________________________________________________________________________________________________________________________________________________________________________________________________________________________________________________________________________________________  ________________________________________________________________________________________________________________________________________________________________________________________________________________________________________________________________________________________________ | | | |
| 8.05 | Where did the baby die | Home  On the way to treatment  Health facility  Traditional healer’s place  Other, please specify …….. | 1  2  3  4  5 |  |
| 8.06 | Did the baby look normal or abnormal at birth? | Normal  Abnormal  Don’t know | 1  2  -99 |  |
| 8.07 | In what way do you think the baby looked abnormal? | ……………………………………………...………………………………………………...……… |  |  |
| 8.08 | Did the baby have bruises or signs of injury? | Yes  No  Don’t know | 1  2  -99 |  |
| 8.09 | When your baby was born did anyone do something to help the baby breathe? | Yes  No  Don’t know | 1  2  -99 |  |
| 8.10 | Who did something to help the baby breathe? | Doctor  Nurse  TBA  Village doctor  Husband  Relative/Friend  Other, please specify .............................. | 1  2  3  4  5  6  7 |  |
| 8.11 | What did they do?  *(Circle all that apply)*  *Stimulation includes holding baby upside down, smacking bottom, flicking feet, etc.)* | Stimulation  Mouth to mouth  Bag and mask  Intubated  Oxygen  Other, please specify……………………..  Don’t know | 1  2  3  4  5  -99 |  |
| 8.12 | When did the baby first breathe or cry? | Very soon after birth (within 1 minute)  Soon after birth (1-5 minutes)  A long time after birth (5-30 minutes)  Never  Don’t know | 1  2  3  4  -99 |  |
| 8.13 | 5 minutes after birth which of these descriptions best describes your baby? | Crying well, breathing well, pink and active  Poor cry, poor breathing, blue limbs, poorly active  No cry, blue body, slow heartbeat, no movement  Don’t know | 1  2  3  -99 |  |
| 8.14 | Were the baby’s arm and legs normal, floppy or stiff? | Normal  Floppy  Stiff | 1  2  3 |  |
| 8.15 | Did the baby have a very small head?(show photo of anencephaly) | Yes  No | 1  2 |  |
| 8.16 | Did the baby have a mass or defect on the back of the head or spine? (show photo of meningomyelocele) | Yes  No | 1  2 |  |
| 8.17 | Did the baby have a cleft lip or palate? (show photo of cleft lip and palate) | Yes  No | 1  2 |  |
| 8.18 | Did the baby have abnormal arms or legs? | Yes  No | 1  2 |  |
| 8.19 | Was the baby sleepy and floppy at the time of birth? | Yes  No | 1  2 |  |
| 8.20 | Did the baby ever have difficulty breathing? | Yes  No | 1  2 | 8.21  8.29 |
| 8.21 | If the baby had difficulty breathing, what was the difficulty? (demonstrate) | Intermittent  Fast breathing | 1  2 |  |
| 8.22 | If the baby had difficulty breathing, when did the difficulty start? | Immediately after birth  Not immediately, but within 6 hours  More than 6 hours after birth  Don’t know | 1  2  3  -99 |  |
| 8.23 | If the baby had difficulty breathing, how long did the difficulty continue? | _____________ days |  |  |
| 8.24 | If the baby had difficulty breathing, did it continue till the baby died? | Yes  No | 1  2 |  |
| 8.25 | If the baby had difficulty breathing, was there chest in drawing?  (demonstrate) | Yes  No | 1  2 |  |
| 8.26 | If the baby had difficulty breathing, did it grunt?(demonstrate) | Yes  No | 1  2 |  |
| 8.27 | If the baby had difficulty breathing, did it flare its nostrils? (demonstrate) | Yes  No | 1  2 |  |
| 8.28 | If the baby had difficulty breathing, did the baby have pneumonia? | Yes  No | 1  2 |  |
| 8.29 | Did the baby ever suckle normally? | Yes  No  Don’t know | 1  2  -99 | 8.30  8.31  8.36 |
| 8.30 | Did the baby always suckle normally? | Yes  No  Don’t know | 1  2  -99 | 8.37  8.31 |
| 8.31 | If the baby did not always suckle normally, when did the problem start? | On the 1^st^ day  After the 1^st^ day but in the 1^st^ 3 days  After the 1^st^ 3 days  Don’t know | 1  2  3  -99 |  |
| 8.32 | If the baby did not always suckle normally, for how long did the problem continue? | ______________days  Don’t know | -99 |  |
| 8.33 | If the baby did not always suckle normally, did the feeding problem continue until the baby died? | Yes  No  Don’t know | 1  2  -99 |  |
| 8.34 | If the baby did not always suckle normally, could the baby open his/her mouth? | Yes  No | 1  2 |  |
| 8.35 | If the baby did not always suckle normally, did the baby arch his/her back and have spasms?(show picture) | Yes  No | 1  2 |  |
| 8.36 | If the baby did not always suckle normally, did the baby have tetanus? | Yes  No | 1  2 |  |
| 8.37 | Was the baby’s stool more liquid than normal? | Yes  No | 1  2 |  |
| 8.38 | Did the baby pass stool more frequently than normal? | Yes  No | 1  2 |  |
| 8.39 | If the baby had diarrhoea, was there mucus or blood in the stool? | Yes  No | 1  2 |  |
| 8.40 | If the baby had diarrhoea, how long did it continue? | _______________days |  |  |
| 8.41 | Did the baby vomit everything? | Yes  No | 1  2 |  |
| 8.42 | Did the baby have fever? | Yes  No | 1  2 | 8.43  8.45 |
| 8.43 | If the baby had fever, how many days after birth did the fever start? | _____________days |  |  |
| 8.44 | If the baby had fever, for how long did the fever continue? | ________________days |  |  |
| 8.45 | Were the baby’s skin and eyes very yellow? | Yes  No | 1  2 |  |
| 8.46 | Did the baby have any seizures/ fits/convulsions for the first time? | Yes  No | 1  2 | 8.47  8.51 |
| 8.47 | On which day of life did the baby have fits/seizures? | First day  After first day |  |  |
| 8.48 | Did the baby feel cold? | Yes  No | 1  2 | 8.49  8.51 |
| 8.49 | If the baby felt cold, at how many days after birth did the baby start feeling cold? | _______________days |  |  |
| 8.50 | If the baby felt cold, for how long did the baby feel cold? | ______________Hours  ______________Days |  |  |
| 8.51 | Did the baby have pustules on the skin? | Yes  No | 1  2 |  |
| 8.52 | Did the baby have ear discharge? | Yes  No | 1  2 |  |
| 8.53 | Did the baby have red eyes with pus in them? | Yes  No | 1  2 |  |
| 8.54 | Did the baby have a bright red ring on the skin around the umbilicus cord stump? | Yes  No | 1  2 |  |
| 8.55 | Did the baby bleed? | Yes  No | 1  2 | 8.56  8.57 |
| 8.56 | From where did the baby bleed? | ___________________________________________________________________________ |  |  |
| 8.57 | What did the baby’s fontanelle look like?(point to fontanelle) | Sunken down  Normal  Bulging up |  |  |
| 8.58 | Did the baby become drowsy and unconscious when it had been normal before? | Yes  No | 1  2 |  |
| 8.59 | How long was the baby ill before he/she died? | ____________days |  |  |
| 8.60 | Did the baby die without any sign of illness? | Yes  No | 1  2 |  |
| 8.61 | Did the baby have some other problem that we have not discussed? | Yes  No | 1  2 | 8.62  8.63 |
| 8.62 | If Yes, what problem? | ___________________________________________________________________________ |  |  |
| 8.63 | Do you think the death could have been prevented? | Yes  No  Don’t know | 1  2  -99 | 8.64  END  END |
| 8.64 | How do you think the death could have been prevented? | _________________________  ____________________________________________________________________________________________________ |  |  |

***Thank you very much for your cooperation.***

| **Mother ID** | | | | |
| --- | --- | --- | --- | --- |
|  |  |  |  |  |

**SECTION 9: MATERNAL VERBAL AUTOPSY (Relative)**

**I would like to ask you some questions to try to help me understand exactly how and why the mother died. I understand that this is difficult for you to talk about and I am very grateful for any information that you are able to provide.**

Initials____________________________________________ Relationship to the deceased_______________

Date______________________

| **No** | **Question** | **Responses** | **Code** | | **Go to** | |
| --- | --- | --- | --- | --- | --- | --- |
| 9.01 | Were you present with the mother when she died? | Yes  No | 1  2 | | 9.03  9.02 | |
| 9.02 | How long before her death did you see her? | < 6 hours  6-12 hours  12-24 hours  1-3 days  4-7 days  > 1 week | 1  2  3  4  5  6 | |  | |
| 9.03 | Who was present with the mother when she died?  *(Circle all that apply)* | TBA  Health care provider  Her husband  Her mother  Her mother-in-law  No one  Other, please specify________________  _________________________________ | 1  2  3  4  5  6  7 | | 9.04  9.04  9.05 | |
| 9.04 | If a TBA or health care provider was present what is her name? | __________________________________________________________________ |  | |  | |
| 9.05 | I would really appreciate it if you could help me to try to understand exactly how and why she died. Please tell me in detail all of the problems that occurred from when she became ill until she died. Please tell me everything that happened even if it means repeating some of the information that you have already provided. At the end please also say what you consider to be the cause of death. *This should be recorded as spoken by the respondent.*  ________________________________________________________________________________________________________________________________________________________________________________________________________________________________________________________________________________________________________________________________________________________________________________________________________________________________________________________________________________________________________________________________________________________________________________________________________________________________________________________________________________________________________________________________________________________________________________________________________________________________________________________________________________________________________________________________________________________________________  Interviewer try to classify into one of these (circle number):  1. Related to termination of pregnancy  2. Antepartum Haemorrhage  3. Eclampsia  4. Postpartum Haemorrhage 9.07  5. Sepsis  6. Obstructed labour / ruptured uterus / shock  7 Other, please specify……….  8. Accidental death 9.06 | | | | | |
| 9.06 | Did the mother die due to any of the following? | Traffic accident  Drowning  Burns  Poisoning  Firearms, weapon accident  Suicide  Homicide  None of these | 1  2  3  4  5  6  7  8 | |  | |
| 9.07 | Before the mother became pregnant the last time did she have any sort of illness? | Yes  No  Don’t know | 1  2  -99 | |  | |
| 9.08 | What sort of illness? | ___________________________________________________________________________________________________ |  | |  | |
|  |  |  | **Y** | **N** | |  |
| 9.09 | Did she suffer from any of the following before she became pregnant? | 1.Tuberculosis  2.Diabetes  3.High blood pressure  4.Jaundice  5.Heart Disease  6.Goitre  7.Kidney disease  8.Epilepsy  9.Malaria | 1  1  1  1  1  1  1  1  1 | 2  2  2  2  2  2  2  2  2 | |  |
| 9.10 | Had she ever had any of the following as a result of previous pregnancies? | Miscarriage  Stillbirth  Premature birth  Caesarean operation to deliver baby  Not applicable  None of the above | 1  1  1  1  1  1 | 2  2  2  2  2  2 | |  |
| 9.11 | What was the date of the mother’s death? | _____/______/____ (dd/mm/yy)  Don’t know | -99 | |  | |
| 9.12 | When did her death occur? | During early pregnancy, before the sixth month  After the sixth month but before labour began  During labour or delivery  After delivery but within the following 6 weeks  More than 6 weeks but less than one year after delivery  Don’t know | 1  2  3  4  5  -99 | | 9.13  9.15  9.15  9.13 | |
| 9.13 | How many months pregnant was the mother when she died? | _______months  Don’t know |  | |  | |
| 9.14 | Did she die with the baby still inside her? | Yes  No  Don’t know | 1  2  -99 | |  | |
| 9.15 | Where did she die? | At a health facility  On the way to a health facility  At home  Other, please specify ................................ | 1  2  3  4 | | 9.16  9.17 | |
| 9.16 | Name of health facility | _________________________________ |  | |  | |
| 9.17 | Did the mother or anyone decide to seek care before she died? | Yes  No | 1  2 | |  | |
| 9.18 | If no, why did no one decide to seek care?  *(Circle all that apply)* | Did not see a need to  Did not know where to go  Provider is too far away  No transport to reach provider  Roads were bad  No one to accompany  Too busy to go to provider  Could not afford to go to provider  Family forbade to visit provider  No one able to look after children  Facility has inconvenient opening times  Inconvenient timings of nurse’s visits  Facility / provider has poor quality service  Did not like attitude of staff  No provider available at facility  Other, please specify………………………. | 1  2  3  4  5  6  7  8  9  10  11  12  13  14  15  16 | | 9.34    9.34 | |
| 9.19 | For which problem(s) did you decide to seek care? | 1……………………………………………  2……………………………………………  3……………………………………………  4……………………………………………  5…………………………………………… |  | |  | |
| 9.20 | How long was it from the onset of the illness until someone decided to seek care? | Immediately  < 6 hours  6-12 hours  12-24 hours  > 24 hours, please specify…………..days  Don’t know | 1  2  3  4  5  -99 | |  | |
| 9.21 | Where was care sought first? | Home visit  PHCU/ HC II  Primary health centre/ PHCC  District Hospital  NGO facility  Private facility  At provider’s home  Other, please specify ........................….. | 1  2  3  4  5  6  7  8 | | 9.22  9.23 | |
| 9.22 | If care was received at home, how long was it from when someone decided to seek care until the mother received care? | Immediately  < 6 hours  6-12 hours  12-24 hours  > 24 hours, please specify………….days  No care was received  Don’t know | 1  2  3  4  5  6  -99 | | 9.26 | |
| 9.23 | After deciding to seek care how long was it before the mother arrived at the health facility? | Immediately  < 6 hours  6-12 hours  12-24 hours  > 24 hours, please specify………….days  Don’t know | 1  2  3  4  5  -99 | |  | |
| 9.24 | After arriving at the health facility how long was it before a member of staff provided care for the mother? | Immediately  < 6 hours  6-12 hours  12-24 hours  > 24 hours, please specify………….days  Don’t know  No care was received | 1  2  3  4  5  -99  -88 | | 9.26  9.25 | |
| 9.25 | Why did the mother receive no care?  *(Circle all that apply)* | Facility closed  Facility had inadequate supplies / equipment  Staff too busy  Staff not available  Staff refused to see  Other, please specify………………………  ………………………………………………. | 1  2  3  4  5  6 | | 9.28  9.28 | |
| 9.26 | Who examined her? | Doctor  Nurse  TBA  Village doctor  Relative/Friend  Other, please specify .............................. | 1  2  3  4  5  6 | |  | |
| 9.27 | What treatment did she receive? | ___________________________________________________________________________________________________ |  | |  | |
| 9.28 | Was the mother referred to another facility? | Yes  No | 1  2 | | 9.29  9.32 | |
| 9.29 | What facility was she referred to? | Primary health centre  District Hospital  Charitable hospital  NGO facility  Private facility  Other, please specify ………………….. | 1  2  3  4  5  6 | |  | |
| 9.30 | Did she go there? | Yes  No | 1  2 | | 9.32  9.31 | |
| 9.31 | Why did she not go there?  *(Circle all that apply)* | Did not see a need to  Did not know where to go  Did not know about antenatal care  Provider is too far away  No transport to reach provider  No one to accompany  Too busy to go to provider  Could not afford to go to provider  Family forbade to visit provider  No one able to look after children  Facility has inconvenient opening times  Facility / provider has poor quality service  Did not like attitude of staff  No provider available at facility  Other, please specify………. | 1  2  3  4  5  6  7  8  9  10  11  12  13  14  15 | |  | |
| 9.32 | Did she experience any difficulties while trying to access care? | Yes  No | 1  2 | | 9.33  9.34 | |
| 9.33 | What difficulties did she experience while trying to access care?  *(Circle all that apply)* | Did not receive any treatment  Had to wait a long time for treatment  Facility had inadequate supplies / equipment  Did not see provider  Staff were not adequately qualified  Staff were rude  Transport problems to reach facility  Expensive / could not afford  Facility was closed  Other, please specify………………………  ………………………………………………. | 1  2  3  4  5  6  7  8  9  10 | |  | |
| 9.34 | Do you think she could have been saved? | Yes  No | 1  2 | | 9.35  END | |
| 9.35 | Please explain how you think she could have been saved…  __________________________________________________________________________________________________________________________________________________________________________________________________________________________________________________________________________________________________________________________________________________________________________________________________________________________________________________________________________________________________________________________________________________________________________________________________________________________________________________________________________________________________________________________________________________________________________________________________________________________________________________________________________________________ | | | | | |

***Thank you very much for your cooperation.***

| **Mother ID** | | | | |
| --- | --- | --- | --- | --- |
|  |  |  |  |  |

**SECTION 10: MATERNAL VERBAL AUTOPSY (Provider)**

**I would like to ask you some questions to try to help me understand exactly how and why the mother died. I understand that this is difficult for you to talk about and I am very grateful for any information that you are able to provide.**

| **No** | **Question** | **Responses** | **Code** | **Go to** |
| --- | --- | --- | --- | --- |
| 10.01 | Were you present with the mother when she died? | Yes  No | 1  2 | 10.03  10.02 |
| 10.02 | How long before her death did you see her? | < 6 hours  6-12 hours  12-24 hours  1-3 days  4-7 days  > 1 week | 1  2  3  4  5  6 |  |
| 10.03 | Who was present with the mother when she died?  *(Circle all that apply)* | TBA  Health care provider  Her husband  Her mother  Her mother-in-law  No one  Other, please specify________________  _________________________________ | 1  2  3  4  5  6  7 |  |
| 10.04 | I would really appreciate it if you could help me to try to understand exactly how and why she died. Please tell me in detail all of the problems that occurred from when she became ill until she died. Please tell me everything that happened even if it means repeating some of the information that you have already provided. At the end please also say what you consider to be the cause of death. *This should be recorded as spoken by the respondent.*  _______________________________________________________________________________________________________________________________________________________________________________________________________________________________________________________________________________________________________________________________________________________________________________________________________________________________________________________________________________________________________________________________________________________________________________________________________________________________________________________________________________________________________________________________________________________________________________________________________________________________________________________________________________________________________________________________________________________________________________________________________________  Interviewer try to classify into one of these (circle number):  1. Related to termination of pregnancy  2. Antepartum Haemorrhage  3. Eclampsia  4. Postpartum Haemorrhage 10.06  5. Sepsis  6. Obstructed labour / ruptured uterus / shock  7 Other, please specify......................................................  8. Accidental death 10.05 | | | |
| 10.05 | Did the mother die due to any of the following? | Traffic accident  Drowning  Burns  Poisoning  Firearms, weapon accident  Suicide  Homicide  None of these | 1  2  3  4  5  6  7  8 |  |
| 10.06 | What was the date of the mother’s death? | _____/______/____ (dd/mm/yy)  Don’t know | -99 |  |
| 10.07 | When did her death occur? | During early pregnancy, before the sixth month  After the sixth month but before labour began  During labour or delivery  After delivery but within the following 6 weeks  More than 6 weeks but less than one year after delivery  Don’t know | 1  2  3  4  5  -99 | 10.08  10.10  10.08 |
| 10.08 | How many months pregnant was the mother when she died? | _______months  Don’t know |  |  |
| 10.09 | Did she die with the baby still inside her? | Yes  No  Don’t know | 1  2  -99 |  |
| 10.10 | Where did she die? | At a health facility  On the way to a health facility  At home  Other, please specify ................................ | 1  2  3  4 | 10.11  10.12 |
| 10.11 | Name of health facility | _________________________________ |  |  |
| 10.12 | Did you provide any treatment for the mother? | Yes  No | 1  2 | 10.13  10.14 |
| 10.13 | What treatment did she receive? | ___________________________________________________________________________________________________ |  |  |
| 10.14 | Why did you not provide any treatment? | ___________________________________________________________________________________________________ |  |  |
| 10.15 | Was the mother referred to another facility? | Yes  No | 1  2 | 10.16  10.17 |
| 10.16 | What facility was she referred to? | Primary health centre  District Hospital  Charitable hospital  NGO facility  Private facility  Other, please specify……………….. | 1  2  3  4  5  6 |  |
| 10.17 | Do you think she could have been saved? | Yes  No | 1  2 | 10.18  END |
| 10.18 | Please explain how you think she could have been saved…  ________________________________________________________________________________________________________________________________________________________________________________________________________________________________________________________________________________________________________________________________________________________________________________________________________________________________________________________________________________________________________________________________________________________________________________________________________________________________________________ | | | |

***Thank you very much for your cooperation.***
